# Integrative profiling of gene expression and chromatin accessibility elucidates specific transcriptional networks in porcine neutrophils

**DOI:** 10.3389/fgene.2023.1107462

**Published:** 2023-05-23

**Authors:** Juber Herrera-Uribe, Kyu-Sang Lim, Kristen A. Byrne, Lance Daharsh, Haibo Liu, Ryan J. Corbett, Gianna Marco, Martine Schroyen, James E. Koltes, Crystal L. Loving, Christopher K. Tuggle

**Affiliations:** ^1^ Department of Animal Science, Iowa State University, Ames, IA, United States; ^2^ Department of Animal Resource Science, Kongju National University, Yesan, Republic of Korea; ^3^ USDA-Agriculture Research Service, National Animal Disease Center, Food Safety and Enteric Pathogens Research Unit, Ames, IA, United States

**Keywords:** pig, transcriptome, bulk-RNA-seq, co-expressed genes, transcription factor (TF), gene regulation, immune cells, ATAC-seq

## Abstract

Neutrophils are vital components of the immune system for limiting the invasion and proliferation of pathogens in the body. Surprisingly, the functional annotation of porcine neutrophils is still limited. The transcriptomic and epigenetic assessment of porcine neutrophils from healthy pigs was performed by bulk RNA sequencing and transposase accessible chromatin sequencing (ATAC-seq). First, we sequenced and compared the transcriptome of porcine neutrophils with eight other immune cell transcriptomes to identify a neutrophil-enriched gene list within a detected neutrophil co-expression module. Second, we used ATAC-seq analysis to report for the first time the genome-wide chromatin accessible regions of porcine neutrophils. A combined analysis using both transcriptomic and chromatin accessibility data further defined the neutrophil co-expression network controlled by transcription factors likely important for neutrophil lineage commitment and function. We identified chromatin accessible regions around promoters of neutrophil-specific genes that were predicted to be bound by neutrophil-specific transcription factors. Additionally, published DNA methylation data from porcine immune cells including neutrophils were used to link low DNA methylation patterns to accessible chromatin regions and genes with highly enriched expression in porcine neutrophils. In summary, our data provides the first integrative analysis of the accessible chromatin regions and transcriptional status of porcine neutrophils, contributing to the Functional Annotation of Animal Genomes (FAANG) project, and demonstrates the utility of chromatin accessible regions to identify and enrich our understanding of transcriptional networks in a cell type such as neutrophils.

## Introduction

Neutrophils originate from myeloid progenitors in the bone marrow and go through numerous phases of development, including myeloblast, promyelocyte, myelocyte, metamyelocyte, band cell, and eventually polymorphonuclear neutrophil (Hong, 2017). Neutrophils are one of the most common and important immune cell types in the vertebrate bloodstream (Fingerhut et al., 2020); in human they represent 50%–70% of all leukocytes (Mortaz et al., 2018). Neutrophils are recruited to areas of inflammation from the bloodstream, migrating through chemical signal gradients in a process known as chemotaxis (de Oliveira et al., 2016). Neutrophils are among the most specific immune cell responding to chemotaxic signals, and serve as an overarching model for eukaryotic chemotaxis (Metzemaekers et al., 2020a). Neutrophils respond to chemoattractant signals by secreting granules (degranulation), ingesting microorganisms and other particles (phagocytosis), and forming neutrophil extracellular traps that catch and kill extracellular bacteria once they arrive at the inflammation site (Gierlikowska et al., 2021). Importantly, neutrophils are among the most fully differentiated immune cells (Hong, 2017) and the resting state is highly informative for neutrophil function. Thus, multiple pathways, many unique to the neutrophils, are fully deployed in non-activated neutrophils and the transcription regulation to effect neutrophil activity is poorly understood in the pig.

Genome-wide transcriptomic approaches can identify transcriptional signatures in whole blood (Liu et al., 2017) and specific populations of porcine immune cells (Herrera-Uribe et al., 2021), including neutrophils (Huang et al., 2020). Transcriptomic analyses have also been employed to explore different immune populations in pigs under different conditions such as infections, differentiation, tissue niche, and health status (do Nascimento et al., 2018; Dong et al., 2021; Herrera-Uribe et al., 2020; Summers et al., 2020). Recently, bulk RNA sequencing (bulk RNA-seq) and single-cell RNA sequencing (scRNA-seq) was used to identify genes expressed in different subsets and co-expressed clusters of porcine immune cells from peripheral mononuclear cells (PBMC) (Herrera-Uribe et al., 2021). However, as PBMC preparations exclude neutrophils, there is still limited information cataloging the porcine neutrophil transcriptome. So far, only one RNA-seq-based transcriptomic study (Huang et al., 2020) and two array-based studies (Sanz-Santos et al., 2011; Wang et al., 2017) have been published, but no study exploring the regulatory elements involved in the transcriptional network controlling such RNA expression patterns has been reported.

Genome-wide chromatin accessibility assays can identify genomic regions physically accessible to transcriptional machinery and provide clues to cell-specific gene expression mechanisms that determine the cell identity and function (Yan et al., 2020). For example, recruitment of regulatory proteins to specific transcription factor binding motifs (TFBM) found in such accessible regions provides the opportunity to activate or maintain specific cellular functions (Stein et al., 2010). Therefore, identifying open chromatin regions helps to identify possible regulatory elements and predict cell functions and regulatory pathways in different cell types (Natarajan et al., 2012). Several epigenomic assays can identify regulatory regions genome-wide, such as the DNase I hypersensitivity assay (Pipkin and Lichtenheld, 2006), and more recently the Assay for Transposase Accessible Chromatin (ATAC-seq) (Buenrostro et al., 2013). Combining epigenetic assays, such as ATAC-seq with gene expression assays such as RNA-seq, provides a powerful approach to identify accessible chromatin regions genome-wide, associates the pattern of such regions with RNA expression, and establishes transcriptional regulatory networks to identify specific regulatory functions (Lowe et al., 2019).

A combined genome-wide annotation of chromatin accessibility and transcriptome of porcine neutrophils will contribute to the functional annotation of the porcine genome (part of the FAANG project), enriching our understanding of the molecular regulation of neutrophils, and provide tools for increased utilization of the pig as a model to explore human immunity. In this study, we used ATAC-seq to identify open chromatin regions, and RNA-seq to describe the specific gene expression pattern in porcine neutrophils through comparison to published transcriptomic data of eight other immune cell types that were magnetically and flow cytometrically sorted from peripheral blood mononuclear cells (Myeloid, NK, and several populations of T and B cells) of the same animals. In summary, our analysis revealed co-expressed and specific transcriptional patterns in porcine neutrophils, including transcription factors that were predicted to bind to accessible promoter regions of neutrophil-specific genes (NSGs).

## Materials and methods

### Animals and peripheral blood mononuclear cell isolation

Two crossbred (predominantly Large White and Landrace heritage) male pigs of 6 months of age were used for RNA-seq and ATAC-seq, and four crossbred adults (>6 months of age, unknown sex) were used for Fluidigm Reverse Transcriptase Polymerase Chain Reaction (RT-qPCR) validation. Pigs were housed in BLS2 rooms at the National Animal Disease Center (Ames, IA) and all animal procedures were performed in compliance with and approval by Institutional Animal Care and Use Committee. Pigs were observed daily by animal care staff and were bright, active, and alert during their life. No veterinary treatment was warranted the month prior to sample collection. Upon necropsy, no gross pulmonary lesions suggestive of pneumonia were noted. Up to 120 mL of blood was drawn into BD Vacutainer mononuclear Cell Preparation Tubes—Sodium Citrate (CPTTM, Becton Dickson) or 60-mL syringes containing 6 mL of acid citrate dextrose (ACD). PBMCs were isolated, enumerated, and viability assessed as previously described (Byrne et al., 2021).

### Neutrophil isolation

Neutrophils were isolated from whole blood in CPTTM tubes as previously described (Heit et al., 2006; Lindblom et al., 2018). Briefly, whole blood was subjected first to dextran sedimentation using 6% Dextran/0.9% NaCl solution at room temperature for 45-60 min. Supernatant containing neutrophils and mononuclear cells was transferred to a conical tube and centrifuged for 12 min at 300 RCF, 4°C with low brake. Neutrophils and mononuclear cells were separated from the erythrocyte-rich pellet by lysing erythrocytes with Ammonium-Chloride-Potassium (ACK) Lysing buffer (ThermoFisher). The pellet was resuspended in phosphate buffered saline (PBS) and the cell suspension was layered over Ficoll-Histopaque-1077 (Catalog No.1077, Sigma) and centrifuged for 30 min at 450 RCF, room temperature, low brake. After centrifugation, PBMCs were removed, and the pellet that contained neutrophils was further processed. Neutrophils were rinsed with 4 mL of Hanks’ Balance Salt Solution (HBSS), centrifuged at 450 RCF for 5 min and resuspended in 2 mL HBSS. Cell count and viability data were obtained using the MUSE cell analyzer system (Millipore). Cells were cryopreserved using 30% dimethyl sulfoxide (DMSO) in RPMI medium and thawed for later use following a previously reported protocol (Milani et al., 2016). A flowchart of the methods is shown in the Supplementary Figure S1.

### RNA isolation for bulk RNA-seq and fluidigm analysis

Isolated neutrophils were enumerated and immediately lysed in RLT-plus buffer for bulk RNA sequencing (bulkRNA-seq). RNA extraction for RLT-plus samples was performed using the AllPrep DNA/RNA MiniKit (QIAGEN) following manufacturer’s instructions. Eluted RNA was treated with RNase-Free DNase (QIAGEN). RNA quantity and integrity were assessed with the Agilent 2200 TapeStation system (Agilent Technologies). RNA samples with RNA integrity numbers (RINs) ≥ 8.2 were used for bulk RNA-seq. Neutrophil RNA extraction for Fluidigm RT-PCR validation were performed using PowerLyzer UltraClean Tissue and Cells RNA Isolation Kit with On-Spin Column DNase I Kit according to manufacturer’s instructions (MoBio, Carlsbad, CA) according to kit instructions.

### Bulk RNA sequencing

RNA was fragmented and prepared into libraries using the TruSeq Stranded Total RNA Sample Preparation Kit (Illumina). The two neutrophil libraries were diluted and pooled in approximately equimolar amounts with other eight sorted immune cells previously reported (Herrera-Uribe et al., 2021), Pooled libraries were sequenced in paired-end mode (2 × 150-bp reads) using an Illumina NextSeq 500 sequencer (300 cycle kit).

### Preprocessing, mapping, alignment and sample level quality control for bulkRNA data

RNA-seq data processing was performed as reported (Herrera-Uribe et al., 2020). The GTF file for the pig reference genome Sscrofa11.1 (Ensembl, version 97) was used as the genome annotation file to be consistent with the previous report, and because version 97 may be more complete for some important immune genes than later versions (Herrera-Uribe et al., 2021). Mapped read counts per gene from pair-end reads and single-end reads generated from initial trimming of each sample were added together to get the final count table. Given that different types of immune cells have quite different transcriptome profiles (Hicks and Irizarry, 2015), YARN (Paulson et al., 2017), a tissue type-aware RNA-seq data normalization tool, was used to filter and normalize the count table. Genes with extremely low expression levels were filtered out using the filterLowGenes function such that only genes with more than 4 read counts in at least one replicate were kept. The final count table contained 10,974 genes, which was then normalized using the normalizeTissueAware function, which leverages the smooth quantile normalization method (Hicks et al., 2018).

Exploratory analysis of RNA-seq data was performed using the DESeq2 package (version 1.24.0) (Love et al., 2014) within the RStudio software (v1.2.1335). First, regularized log-transformation was applied to the normalized count table with the rld function. Then principal component analysis (PCA) and sample similarity analysis was carried out and results were visualized using the plotPCA and distancePlot functions. Heatmaps to display enriched genes were created using the pheatmap package (v1.0.12).

### Identification of cell type-enriched genes in pig bulk RNA dataset

We identified genes with cell type-enriched expression using the method previously reported (Herrera-Uribe et al., 2021). Briefly, a gene was labeled as cell-type enriched if the expression level (averaged across cell types) of a given gene in one cell type was at least two-times higher than the averaged expression level of the given gene across all remaining cell types and the adjusted *p*-value of this contrast was less than 0.05 (Benjamini and Hochberg, 1995). Heatmaps to display enriched genes were created as mentioned above.

For cross species comparison, human and mouse neutrophil (Haemopedia) RNA-seq expression data (Hilton Laboratory at the Walter and Eliza Hall Institute) was used (Choi et al., 2019). Neutrophils for these data sets were isolated using a similar method as described above: depleting red blood cells from the erythrocyte-rich pellet by lysing erythrocytes, plus negative selection of eosinophils and further cell purity confirmation by flow cytometry. Only orthologous genes with one-to-one matches between human and pig (orthologs gene list obtained from Ensembl BioMart (Durinck et al., 2009) were compared. Spearman’s rank correlation analysis was performed to identify correlation between expression levels (transcript per million, TPM) of orthologs gene in pig and human neutrophils. The significance level was set at *p* < 0.05 and the level of Spearman’s rank correlation coefficient (rho, *ρ*) was defined as low (*ρ* < 0.3), moderate (0.3 ≤ *ρ* < 0.5), and strong (*ρ* ≥ 0.5) correlation.

### Gene ontology (GO), protein-protein interaction enrichment analysis (PPI) and transcription factor (TF) enrichment analysis

Gene Ontology (GO) analysis of the top 25% enriched genes was performed using Metascape (Zhou et al., 2019). The threshold *p*-value was set to 0.01. Several terms were clustered into the most enriched GO term. Term pairs with Kappa similarity score above 0.3 were displayed as a network to show relationship among enriched terms. Protein-protein interaction enrichment analysis of the same gene list was performed using STRING (Szklarczyk et al., 2019), BioGrid (Stark et al., 2006), OmniPath (Türei et al., 2016) and InWeb_IM (Li et al., 2017) databases.

The Molecular Complex Detection (MCODE) algorithm was applied to identify densely connected protein network components (Bader and Hogue, 2003). The three best scoring terms by *p*-value were retained as the functional description of the corresponding components. Only orthologous genes were compared. All Ensembl gene IDs with detectable expression level in neutrophils as defined above were used as the background reference. All networks displayed were visualized using Cytoscape.

### Real time PCR verification of differentially expressed genes in neutrophils

Briefly, RT-qPCR analysis was run on the Fluidigm BioMark HD System, using 48 × 48 Fluidigm Dynamic Arrays (Fluidigm, South San Francisco, CA). Primer efficiency was tested simultaneously. Four porcine neutrophils and PBMC RNA samples were included in the assay. Also, serial dilutions of a neutrophil-PBMC pooled sample were included in the Fluidigm plate for primer efficiency calculation. The set of genes assayed were part of a previously developed assay dataset (Schroyen, Marco and Tuggle, unpublished results). Within this independent dataset, we selected all neutrophil significantly enriched genes (SEG, defined below) present (a total of 14) and also selected five genes that were not present in the SEG list for further analysis. The cDNA was pre-amplified for 14 cycles (10 min at 95°C followed by 14 cycles of 95°C for 15 s and 4 min at 60°C) in reactions primed by a master mix of 48 TaqMan Gene Expression Assays (Applied Biosystem) using PreAmp Master Mix (Applied Biosystem). Following a 5-fold dilution of pre-amplified product in water, samples and Assays were loaded onto the plates using the 48.48 MX IFC Controller (Fluidigm). Real-time PCR was performed using the BioMark System for Genetic Analysis (Fluidigm). Cycle threshold (Ct) values were determined using real-time PCR analysis v3.1.3 software (Fluidigm). Ct values corresponding to transcripts encoding *ACTB* and *RPL32* were used as endogenous controls. Changes in transcript expression were calculated using the ΔΔCt method (Livak and Schmittgen, 2001) and converted to log2 scale. Graphs and Student’s t-test statistical analyses were generated using Prism 6 (GraphPad). *p*-values <0.05 were considered statistically significant. Additionally, a Pearson correlation analysis was performed to determine concordance between RNA-seq data and Fluidigm qPCR gene expression values. The significance level for Pearson correlation analysis was set at *p* < 0.05.

### Gene co-expression network analysis

The normalized gene expression levels (transcripts per million; TPM) (Patro et al., 2017) of Significant Enriched Genes (SEGs) (*n* = 832) across 9 cell types were used in the co-expression network analysis using the partial correlation coefficient with information theory (PCIT) algorithm (Reverter and Chan, 2008; Koesterke et al., 2013). The gene co-expression networks of 681 genes (nodes) with the absolute value of the correlation coefficient greater than 0.95 were visualized using Cytoscape v3.8.2 software (Shannon et al., 2003) (Figure 2A). The log2 transformed (TPM+1) values were used to create heatmaps using the pheatmap package in R to show expression patterns across cell types (Figure 2B). To identify candidate neutrophils specific regulators, transcription factors (*n* = 30) in cluster 1 which showed myeloid specific expression were used in another heatmap (Figure 3). The cell expression data from human and mouse (Choi et al., 2019) were plotted into Figure 3 to compare cell type specific expression patterns across species. The gene expression values (TPM) were normalized relative to the gene expression level in neutrophils for comparison purposes across cell types.

### Neutrophil ATAC-seq library preparation

After thawing, neutrophils were immediately lysed, nuclei were counted and transposed with Tn5 transposase following the standard protocol (Buenrostro et al., 2015). In brief, 50,000 nuclei were incubated for 30 min at 37°C in transposase reaction buffer (25 µL TD-2x reaction buffer, 2.5 µL TDR1-Nextera Tn5 Transposase and 22.5 µL Nuclease Free H_2_O). Transposed DNA was isolated using MiniElute PCR Purification Kit (Qiagen) and eluted with 10 µL of Elution buffer (Qiagen). DNA size selection for 100 to 800-bp fragments from the purified libraries was performed using BluePippin cassettes (Sage Science). Libraries were pooled and sequenced on an Illumina HiSeq 3000 sequencer to generate 50-bp paired-end reads at the Iowa State University DNA facility.

### ATAC-seq data analysis

ATAC-seq data processing was performed as previously reported using the nf-core pipeline nf-core/atacseq (Ewels et al., 2020). Read quality was checked using MultiQC version 0.11.7 (Ewels et al., 2016). Illumina sequencing adapters and low-quality bases were trimmed using Trim Galore (version 0.6.5). Paired-end, trimmed reads were separately mapped to the *Sus scrofa* 11.1 pig reference genome using the BWA mem aligner (Li and Durbin, 2010). Sequence duplicates from the paired-end and singleton reads were marked in each individual BAM file, and then BAM files were merged for each sample. Library insert size and duplication rate were checked using picard tools^1^. Duplicate and mitochondrial reads were removed from the aligned reads. The BAM files were used for peak calling with the MACS2 tool, and either broad or narrow peaks were detected with a *q*-value cutoff of 0.05^2^. HOMER was used to annotate peaks relative to known genomic features^3^. The fraction of reads in peaks (FRiP) was calculated using the combined alignments from both replicates. The Integrative Genomics Viewer (IGV) was used to performed visual inspection of the data and visualize the open chromatin regions using bigWig files. Visualization of genome-wide chromatin open regions enrichment at the TSS and across the gene body were visualized using deepTools (Ramírez et al., 2014). Overlapping peaks from all samples were merged to form a consensus peak list.

### Integration of RNA-seq and ATAC-seq data

The read counts of narrow peaks from the ATAC-seq data were standardized as reads per kilobase. The read counts per kilobase were normalized by the trimmed mean of M values (TMM) and then by count per million values, computed for each peak using the EdgeR package in R. An intensity of cut site at open chromatin region (OCR) in the promoter of each gene was assessed by the sum of the normalized count values of the peaks overlapping with a given gene’s promoter region which were defined as the transcription start site (TSS) ± 3 kb. The length of OCRs in the promoter region (TSS-OCRs) for each gene was also calculated. To evaluate the effects of the cut site intensity and the length of accessible regions within promoters on gene expression in neutrophils, Pearson correlations were computed between ATAC-seq peaks either for all expressed genes or for neutrophils enriched genes. The log2 transformed values of gene expression and TSS-OCRs cut intensity and length were used in the correlation analysis.

### Motif enrichment analysis for open chromatin regions in transcriptional start site (TSS-OCR)

To find transcription factors exhibiting neutrophil specific expression, transcription factor binding motifs (TFBM) were identified by enrichment analysis of the neutrophil enriched genes TSS-OCR using the HOMER command findMotifsGenome.pl^4^. The binding motifs for three (*KLF5*, *GFI1B*, and *GATA1*) of the four neutrophil-specific transcription factors identified were annotated in the HOMER database, and these were further investigated to find neutrophil specific genes that had the corresponding motifs in their promoter regions. In addition, protein-protein interactions among neutrophil specific genes were investigated in the STRING database (Szklarczyk et al., 2019). We constructed the network focusing on the four transcription factors identified above with their target genes based on binding motifs and protein interactions.

### Integration of DNA methylation data, RNA-seq and ATAC-seq data

We compared neutrophil gene expression and chromatin accessibility data with previously published DNA methylation data from the same cell populations (Corbett et al., 2022). Average DNA methylation profiles for neutrophil-enriched genes and neutrophil ATAC peaks were generated using deepTools ComputeMatrix command (Ramírez et al., 2016) with default parameters and including 2-kb regions flanking these genes and peaks. Enrichment of neutrophil SEGs and HEGs within neutrophil lowly methylated genes in different genomic feature contexts was calculated using hypergeometric tests. Regions of overlap between neutrophil lowly methylated regions (LMRs) and ATAC peaks were identified using bedtools intersect (Quinlan and Hall, 2010), and overlap enrichment was calculated using hypergeometric tests.

## Results

### Bulk RNA-seq analysis revealed enriched transcriptome gene sets of porcine neutrophils in circulating immune cells and similarities with human neutrophils

The genome-wide transcriptome profile of porcine neutrophils was determined using RNA-seq, and the data was compared with eight different immune cell transcriptomic profiles to identify genes enriched for expression within the neutrophil cell type. RNA-seq libraries contained an average of 40 M clean reads, of which 94.4% aligned to the *Sus scrofa* reference genome after filtering (Supplementary File S1). Relationships among the nine porcine immune cell transcriptomes were assessed by principal component analysis (PCA) and hierarchical clustering (Supplementary Figure S2). While erythrocyte contamination of neutrophils is highly likely in the dataset given the neutrophil isolation method used, we only found low expression of two red blood gene markers (*HBB*: 37 TPM and *HEMGN*: 1 TPM) identified previously by single cell analysis of human red cells (Jain et al., 2022). Read counts for other red blood cell markers such as *HBA*, *HBD*, *HBG*, *NIX*, *ACVR2B*, *AHSP* and *HEMGN* were zero in the porcine neutrophil transcriptome for these samples (Supplementary File S1). These results indicated a low level of erythrocyte contamination in the neutrophil samples, and neither *HBB* nor *HEMGN* were found in further differential gene expression analysis. In the PCA analysis, neutrophils appeared closest to monocytes, and both were clearly distinguished from other cell types.

**FIGURE 1:**
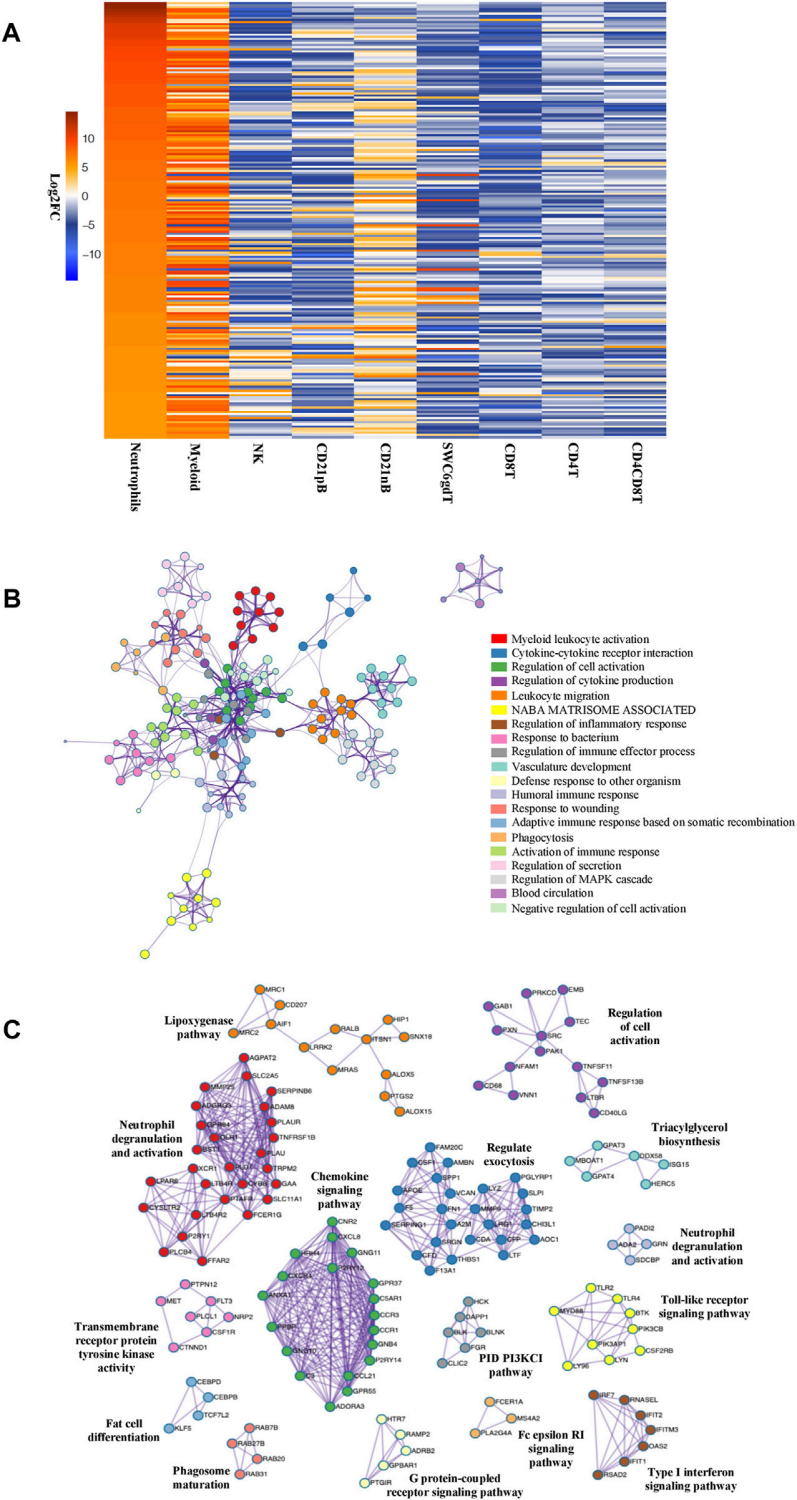
Genes enriched in porcine neutrophil transcriptome are linked to specific GO pathways and PPI networks. **(A)** Heatmap showing the top 25% of HEGs in Neutrophils and eight sorted cells, in decreasing order of fold change difference in expression. **(B)** Ontology enrichment clusters of SEGs. The most statistically significant term within cluster was chosen to represent the cluster. Term color is given by cluster ID and the size of the node is proportional to–log10 *p*-value of enrichment. The stronger the similarity among terms, the thicker the edges between them. **(C)** PPI networks of SEGs. A unique color was assigned to each MCODE network.

The total number of expressed genes detected in neutrophils was 10,974, which were used for differential gene expression (DGE) analysis across cell types. Significantly enriched genes (SEGs) were identified in neutrophils as genes whose expression was both significantly different (adjusted *p*-value<0.05) and a minimum of two-fold higher in expression than the average of all other cell populations (see Methods). In total, we identified 832 SEGs (Supplementary File S1). A subset of the SEGs were selected as highly enriched genes (HEG, 25% highest in log2 FC value) and neutrophil HEG expression across all cell types was visualized (Figure 1A). Although the high expression of these 208 HEG in neutrophils is evident in comparison with the other previously reported cell types, we also observed gene expression similarities with the myeloid (monocytes and dendritic cells) population for many HEG genes (Figure 1A; Supplementary Figure S2). GO analyses using neutrophil HEG indicated enrichment for biological processes such as myeloid leukocyte activation, cytokine-cytokine receptor interaction, response to bacterium and phagocytosis among others, which is characteristic of the neutrophils, and depicted as networks of similar terms (Figure 1B and Supplementary File S2). Protein-protein interaction (PPI) analysis shows proteins in networks that are believed to interact with each other to promote specific biological processes on the basis of experimental and computational data (see Methods). The PPI analysis of HEGs detected 16 subgroups of protein interactions (Figure 1C and Supplementary File S2) such as regulating exocytosis, neutrophil degranulation, type I interferon and NF-kappa B signaling pathways, among others. Overall, evaluation of predicted functional annotations within the neutrophil HEGs provided evidence of genes enriched in functions very consistent with neutrophils, indicating that our isolated cell population and the RNA-seq data obtained represent the expected immune cell type.

Four independent porcine neutrophil and PBMC samples were obtained to validate the RNA-seq expression results. RNA isolation and qPCR analysis of 14 genes was performed using available data from a previously validated assay (Schroyen, Marco and Tuggle, unpublished results). The estimate of fold change ratio calculated by analyzing the genes expression of 19 genes between neutrophil and PBMC samples using qPCR was compared to the fold change ratio of SEGs in neutrophil (see methods) using RNA-seq. In total, fourteen genes within the SEG list (*ALOX5AP*, *BCL6*, *CHI3L1*, *CDF2RB*, *GRN*, *IL13RA1*, *ILR2*, *IL18RAP*, *PLXNC1*, *TLR8*, *TSPAN2*, *SERPING1*, *SOD2* and *VNN2*) and five genes that were not SEGs in neutrophils (*CTSS*, *GMPR*, *PIM1*, *NCF2* and *UBN1*) had gene expression levels that were very consistent with the DGE analysis and confirm the higher expression of SEGs in neutrophils compared to PBMC (Supplementary Figure S3; Supplementary Figure S4). These 19 genes exhibited strong and highly significant positive correlation (rho = 0.71, *p*-value 0.00074) between the RNA-seq and Fluidigm analyses (Supplementary Figure S5). In conclusion, gene expression enrichment found using RNA-seq was confirmed using the Fluidigm qPCR assay in an independent neutrophil dataset.

### Co-expression network and enriched genes reveals specific transcription factors expression in porcine neutrophils

**FIGURE 2:**
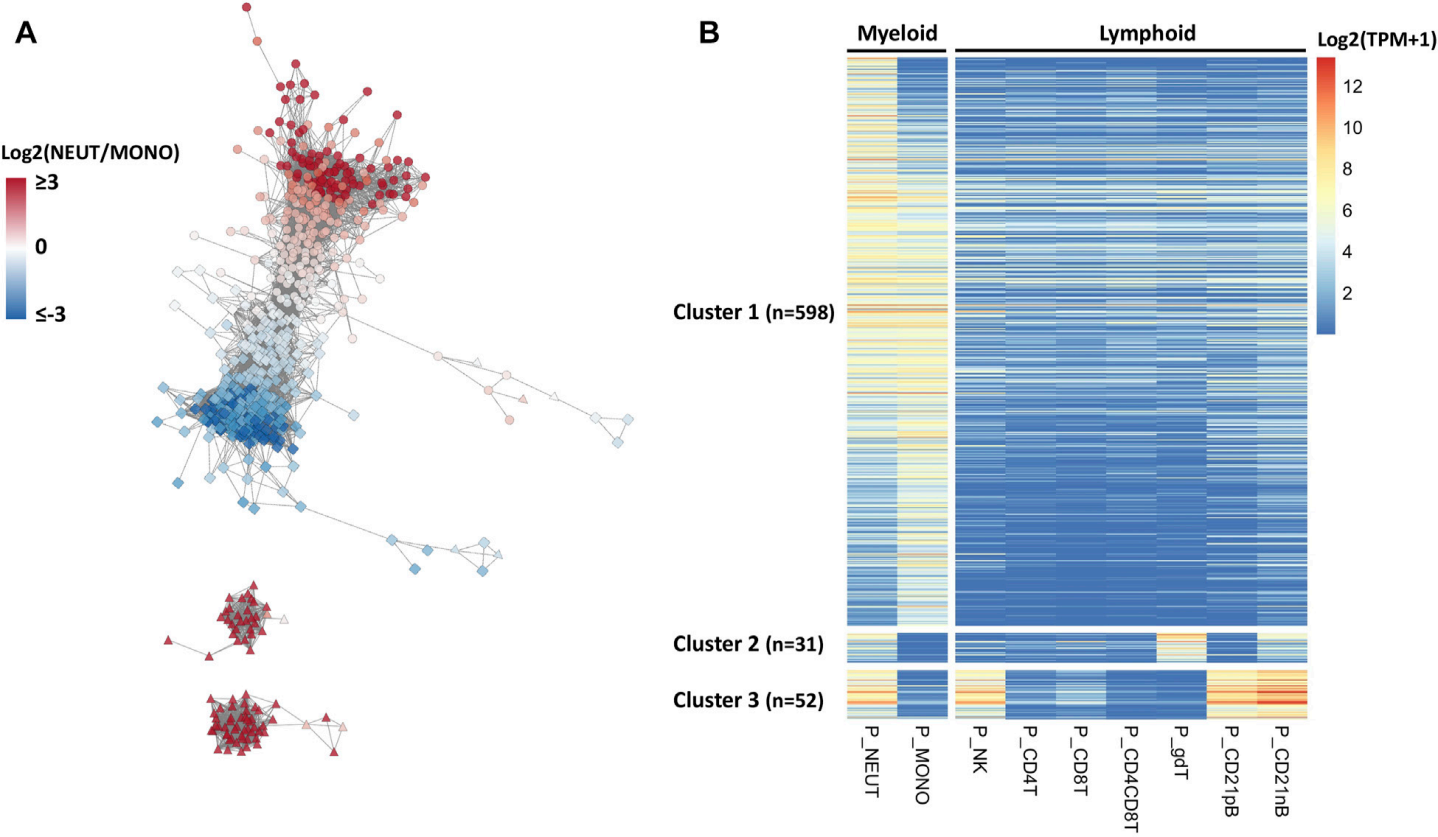
Co-expression network analysis identifying myeloid gene clusters across porcine immune cells. **(A)** Co-expression network analysis (edges between gene nodes shown for correlation r > 0.95) among genes whose expressions were enriched for neutrophils across nine porcine immune cell types and their expression patterns. In the network, the gene nodes (*n* = 681), and node colors indicate log2 fold change (log2FC) between myeloid cells, neutrophils and monotypes (e.g., positive value indicates higher expression in neutrophils). The node shapes represent the cell type with the highest expression for that gene; circle, square, and triangle indicate neutrophils, monotypes, and other cell types, respectively. **(B)** In the heatmap, gene rows were clustered based on the correlation levels and sorted by log2FC in neutrophils.

RNA expression patterns reflect a significant correlation structure in the transcriptome, and co-expressed genes are frequently similar in biological function (van Dam et al., 2018). Hence, we hypothesized that defining co-expressed genes, and identifying those in the co-expressed network that are enriched in neutrophils, would help us to identify specific genes and transcription factors that would be driving the gene expression important for specific porcine neutrophil functions. To address this hypothesis, we defined co-expressed genes across the nine major immune cell type transcriptomes available. Co-expression networks across these cell types contained 681 genes (*r* ≥ 0.95) and were constructed based on the PCIT algorithm (Reverter and Chan, 2008; Koesterke et al., 2013). A co-expression network for all SEGs was constructed from this analysis (Figure 2A). Three easily distinguishable co-expression subnetwork clusters were detected, of which cluster 1 (*n* = 598) showed uniquely higher expressions in myeloid cell types such as neutrophils and monocytes compared to very low expression in lymphoid cell types (Figure 2B and Supplementary File S3). A subset of neutrophil co-expressed genes was also present in the other two clusters that also show expression in other cell types. Genes from cluster 2 genes are expressed in SWC6 (Swine workshop cluster 6) γδ T and CD21 nB B cells, while cluster 3 genes were also expressed in NK cells and B cells (CD21 pB and CD21 nB) (Figures 2A,B, Supplementary File S3).

**FIGURE 3:**
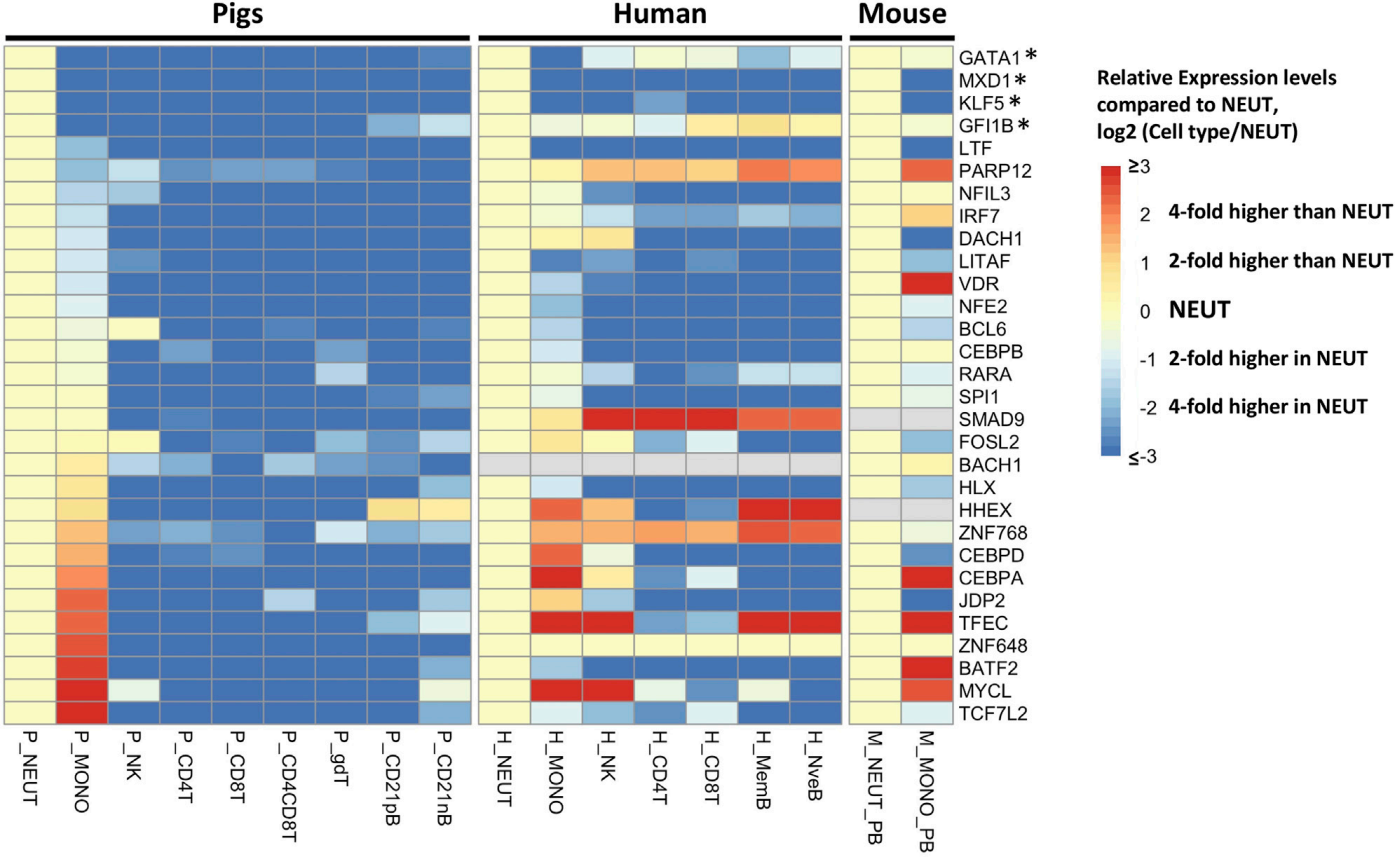
Gene expression pattern of selected transcription factors (*n* = 30) in cluster 1 of the neutrophil network (*r* > 0.95). The human and mouse datasets (Choi et al., 2019) were compared to the pig data, and expression levels across cell types were standardized by the expression levels in neutrophils of each gene to visualize the relative gene expression level compared to that in neutrophils. The asterisk indicates the transcription factors with neutrophil-specific expression in pigs.

To achieve a more refined understanding of the neutrophil biology underlying the co-expression network, we used a more stringent criterion to determine neutrophil-specific genes (NSGs) for further analysis. NSGs were defined as SEGs that were a) co-expressed (*r* ≥ 0.95), b) had highest expression in neutrophils, and c) were statistically significantly (*q* < 0.05) two-fold higher in expression than in monocytes/dendritic cells. A total of 80 NSGs were identified based on these criteria (Supplementary File S3), of which 30 have been annotated as a transcription factor (Figure 3). Four of these NSGs were designated as neutrophil specific transcription factors (NSTF) in pig, and include *GATA1, MXD1, KLF5,* and *GFI1B* (Figure 3). Additionally, the pig neutrophil data allowed us to also identify porcine myeloid specific transcription factors (MSTF) (TF with highest expression in neutrophils or monocytes/dendritic cells and were statistically significantly (*q* < 0.05) two-fold higher in expression than the other seven porcine cell types, Figure 3 and Supplementary File S3). Thus, porcine MSTF were also identified (*DACH1, ZNF648, CEBPB, CEBPA, CEBPD, LTF, LITAF, VDR, NFE2, BATF2, IRF7, TCF7L2, SPI1* and *SMAD9*) (Figure 3). Interestingly, *HHEX* was identified as part of the myeloid co-expression network in Figure 2A but was also highly expressed in B cells; similar TF expression was observed for *FOSL2* and *BCL6* that were part of the myeloid co-expression network and were highly expressed in NK cells.

Finally, we compared the expression of all TFs on the NSGs list with orthologous human and mouse genes from Haemopedia (Choi et al., 2019), finding two NSTF that have higher expression in neutrophil versus monocytes across species, *MXD1* and *KLF5* (Figure 3). Also, *LTF*, *LITAF* and *NFE2* showed higher expression in neutrophils for all three species, but not as high as *MXD1* and *KLF5* in porcine cells. In addition, two MSTF have higher expression in pig and human (*CEBPD*) and in all three species (*CEBPA*).

### Accessible chromatin regions in porcine neutrophils reveals high quality data and moderate correlation with neutrophils enriched genes

**FIGURE 4:**
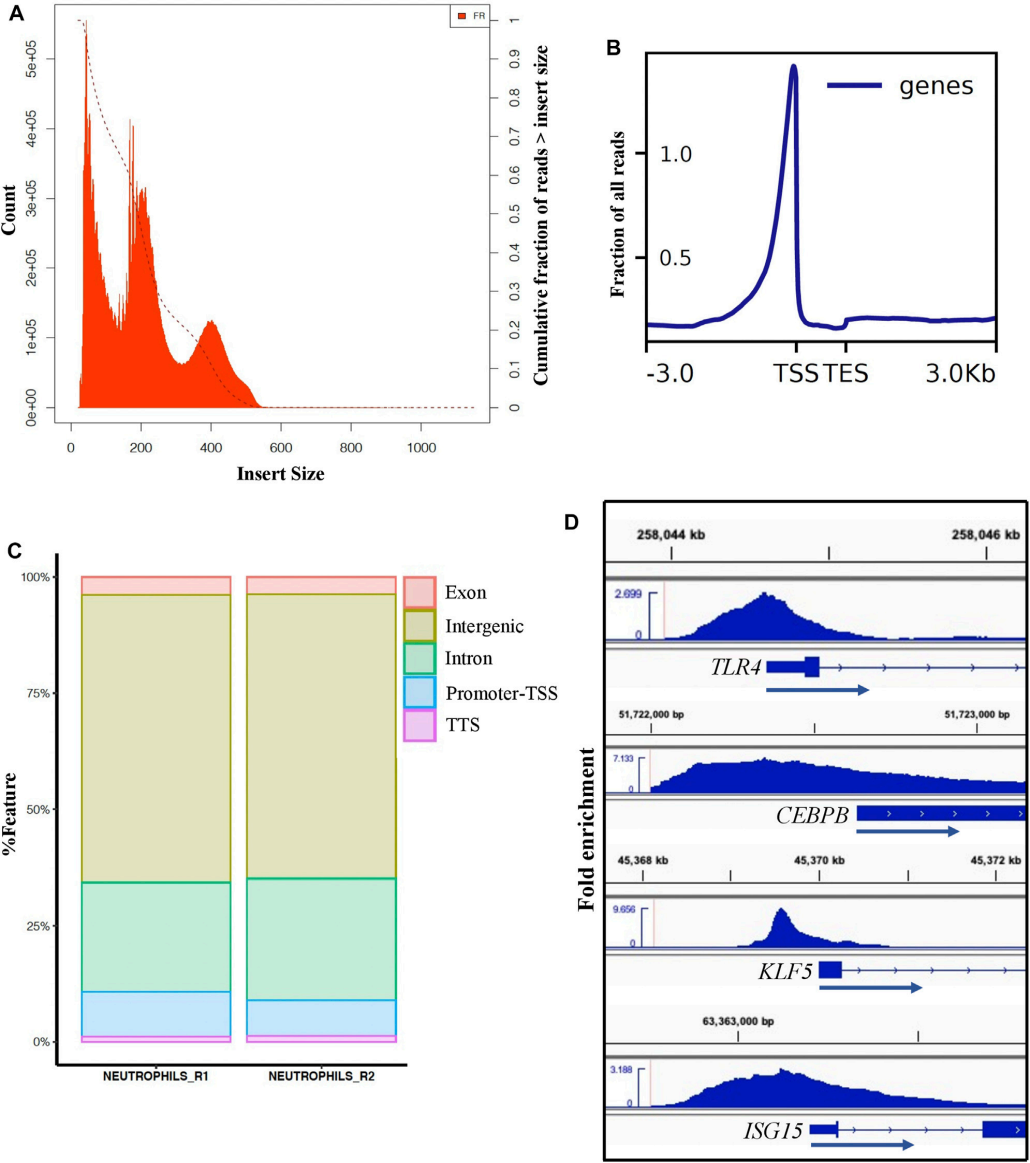
ATAC-seq analysis of porcine neutrophils. **(A)** Distribution of ATAC-seq data fragment length from one neutrophil replicate. Fragment size distribution plot shows enrichment less than 100 bp and around 100-200 bp, indicating nucleosome-free region and mono-nucleosome-bound fragments, respectively. Similar distribution was observed for the other replicate (data not shown). **(B)** TSS enrichment plot from one neutrophil replicate shows that nucleosome-free fragments are enriched at TSS. **(C)** Peak annotation plot shows ATAC-seq peaks distribution genome-wide. **(D)** Visualization of ATAC-seq peaks using the Integrative Genomics Viewer (IGV) near example SEGs in porcine neutrophils (*TLR4* FC: 7.2, *CEBPB* FC: 3, *KLF5* FC: 5.9 and *ISGS15* FC: 4.4).

To define the accessible chromatin in neutrophils from healthy, non-stimulated pigs, we performed ATAC-seq from duplicate samples to those used for neutrophil RNA-seq analysis. We generated approximately 227 million ATAC-seq reads in total, of which 99% were aligned to the *Sus scrofa* reference genome (Supplementary File S4). ATAC-seq libraries contain short fragments (<100 bp), which represent nucleosome-free regions, and larger fragments that span one or more nucleosomes. Clear nucleosome distribution was observed (Figure 4A). Such fragments are mapped to the genome and merged into peaks when sufficient signal is present (Methods). On average, we identified a total of ∼134,000 accessible chromatin elements, of which 57,567 were designed as consensus broad peaks in both library samples meeting the ENCODE standards for ATAC-seq^5^. The ENCODE quality control measure, FRiP score, was over 0.4 in our dataset, which is higher than the minimum of 0.2 used for ENCODE ATAC-seq data (Ou et al., 2018). Consensus narrow peaks averaged around 1 Kb in width, and ATAC-seq signal from both replicates were highly significant and strongly correlated (*r* = 0.93, *p* < 2.2e-16) (Supplementary Figure S6). To confirm and annotate the distribution pattern of the ATAC-seq peaks, we analyzed the ATAC-seq reads distribution across transcriptional start and end sites (TSS, TES) (±3.0 Kb) in *Sus scrofa* reference genome and assigned peaks to genomic features (Figures 4B,C). Although most peaks were located at the intronic or intergenic space (Figure 4C), a clear enrichment of peaks was seen very close to the TSS in the gene-proximal space (Figure 4B), indicating we were able to detect accessible chromatin at active genes. Furthermore, we observed accessible chromatin around TSS regions of enriched genes that are typically involved in pathogen recognition (*TLR4*), DNA-binding transcription activator activity (*CEBPB, KLF5*), and interferon signaling pathway (*ISG15*) (Figure 4D), as well as a lack of accessible chromatin at genes with low or no expression in neutrophils (such as *ZP3* and *FABP6*) (Supplementary Figure S7). These results demonstrate the high sensitivity of ATAC-seq to detect regions associated with active gene promoters.

**FIGURE 5:**
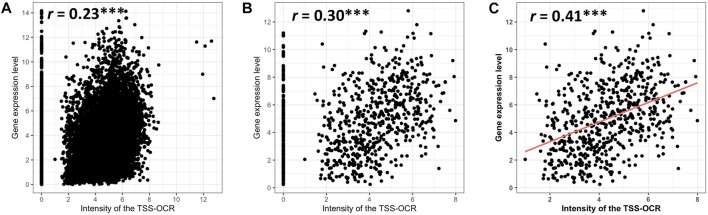
Relationship of gene expression with the intensity of ATAC-seq peak overlapping the gene’s promoter region (TSS-OCR). **(A)** All genes. **(B)** SEGs. **(C)** NSGs that have at least one TSS-OCR (open chromatin regions around ±3 kb from the TSS).

To expand comparison of ATAC-seq signals and gene expression genome-wide, Pearson correlation analysis was performed to test the relationship between gene expression and ATAC-seq signal intensity and length around TSS (±3 kb). Highly significant but relatively low correlation (*r* = 0.23, *p*-value <2.2e-16) was found between the ATAC-signal cut density and all genes used in comparative analysis in this study across cell types (*n* = 11,151) (Figure 5A). Interestingly, similarly significant and moderate correlation (*r* = 0.30, *p*-value <2.2e-16) was observed for enriched genes in neutrophils, (Figure 5B). For both analyses, some expressed genes did not show accessibility within promoter regions, over a range of expression levels. Therefore, we calculated the correlation between enriched genes and accessible regions that had at least one TSS-OCR. As a result, we found the highest correlation with similar significance (*r* = 0.41, *p*-value <2.2e-16) (Figure 5C). Finally, we also identified a weak correlation between gene expression and the length of TSS-OCR in the same comparisons that were performed for ATAC-seq signal intensity (Supplementary Figure S8). Overall, ATAC-seq analysis demonstrated high quality and enrichment for ATAC-seq regions around regulatory regions such as TSS genome-wide.

### Neutrophils DNA methylation is highly negatively associated with RNA-seq and ATAC-seq data

**FIGURE 6:**
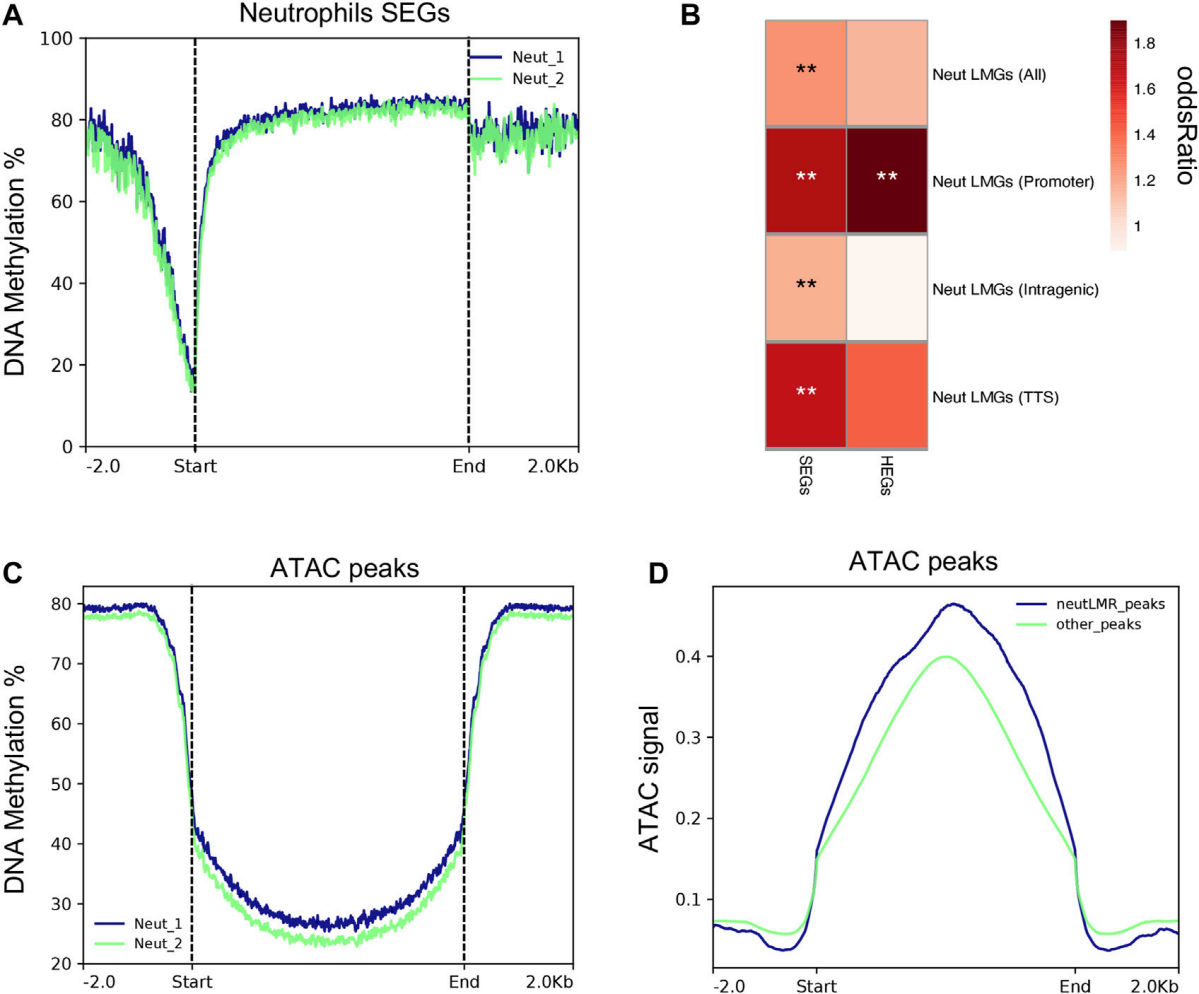
Porcine neutrophil methylation data is associated with neutrophil gene expression and ATAC-seq peaks. **(A)** Methylation rates genome wide across the transcription start site of neutrophils SEGs for each replicate sample. **(B)** Heatmap of normalized enrichment *p*-values of neutrophils lowly methylated regions (LMRs)m SEGs and HEGs overlapping different genomic features. **(C)** Methylation rates genome wide gene across ATAC-seq peaks that were detected in porcine neutrophils. **(D)** Fold enrichment of LMRs genome wide across ATAC-seq peaks.

We used published DNA methylation data generated from samples collected from the same pigs in parallel (Corbett et al., 2022) to determine whether neutrophil DNA methylation was related to gene expression and accessible chromatin regions. Also, we tested the significant enrichment between differentially methylated regions (DMR) (calculated across the same immune cell populations used in this study) and SEGs and HEGs. As is shown in Figure 6A, neutrophils SEGs exhibit low DNA methylation patterns across TSS regions, which indicates activation of gene expression in both neutrophil replicates. Lowly methylated regions (LMRs) were highly enriched among neutrophil SEGs (*p* = 2.4 × 10^-3^). In total, 119 neutrophil SEGs showed LMRs in comparison with other porcine immune cell methylomes reported previously (Supplementary File S6) (Corbett et al., 2022). LMRs across SEGs gene features showed that promoters and transcription termination sires (TTS) have higher enrichment with the expression of SEGs (Figure 6B). However, significant high enrichment of LMRs in intragenic regions of SEGs was also evident, indicating that low methylation was associated with neutrophils SEGs outside promoter regions Figure 6B. The strongest LMR enrichment was observed around promoter of HEGs (*p* = 7.19E-^4^), which are the top 25% of SEGs, demonstrating a stronger association between DNA methylation and gene expression. Additionally, we tested the relationship of genome-wide neutrophil DNA methylation distribution and chromatin accessibility (ATAC-seq signal) across expressed genes in neutrophils. In general, we observed a depletion of DNA methylation in all sequence contexts near open chromatin peaks of expressed genes in neutrophils (Figure 6C). Indeed, we detected 7.4-fold enrichment of LMRs in ATAC-seq peaks (*p* < 1 × 10^-16^), which demonstrate a high association between DNA methylation and chromatin accessibility in porcine neutrophils. There was a trend towards higher ATAC-seq signal among peaks overlapping neutrophil LMRs compared to those not overlapping LMRs (Figure 6D), indicating that, on average, chromatin accessibility increases when coupled with DNA hypomethylation. Collectively, these results link DNA methylation with control of chromatin accessibility and gene expression patterns in porcine neutrophils.

### Transcription factors involved in co-expression networks in porcine neutrophils

**FIGURE 7:**
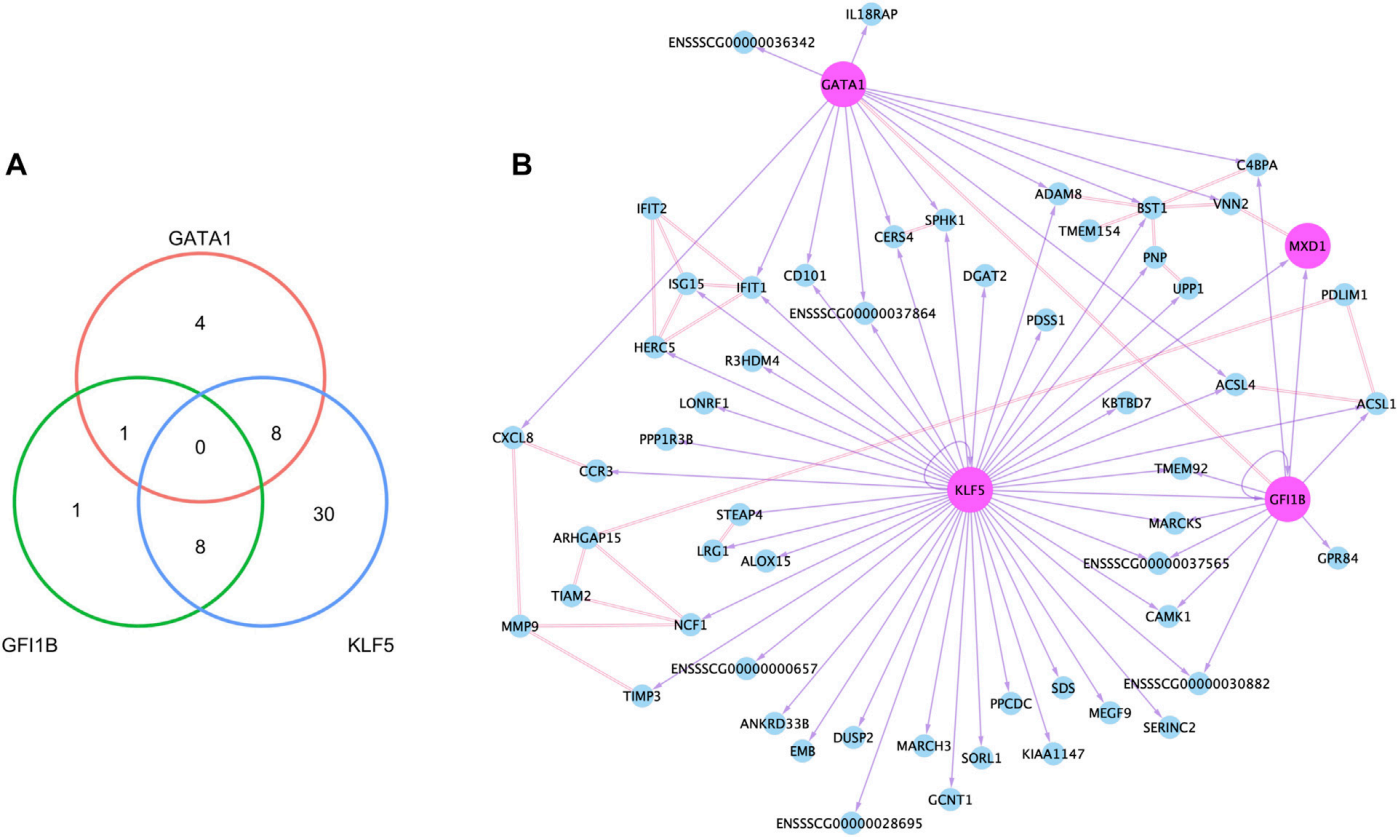
Neutrophil co-expression network created with epigenetic and transcriptomic data. **(A)** The number of genes that had binding motifs for the three neutrophil-specific transcription factors (TFs) within TSS-OCRs, and the network based on binding motifs and protein-protein interactions. In the network **(B)**, the node (circle) indicates each gene, and the pink color indicates TFs. The double line between nodes represents the interaction in the protein level, and the purple arrow with a solid line point to a gene that had a binding motif of the source node.

Accessible chromatin is expected to contain regulatory elements bound by transcription factors controlling expression of nearby and remote genes. To identify the potential regulatory role of NSTFs in NSG promoter regions, we determined the enrichment of transcription factor binding motifs (TFBMs) within narrow ATAC-seq peaks around promoter regions (±3000 bp from TSSs) of NSGs. Within 8,415 peaks around the promoter regions of 7,868 genes expressed in neutrophils, 65 peaks overlapped promoter regions of 58 of the total 80 NSGs (Supplementary Figure S9). TFBM analysis focused on known binding motif on those 65 TSS-OCR revealed that NSTFs such as *GATA1*, *GFI1B*, and *KLF5* were predicted to bind motifs enriched in the TSS-OCR. A total of 52 NSGs were predicted to have motifs within their open chromatin regions corresponding to these NSTF (Figure 7). Most NSGs had predicted enrichment for *KLF5* motifs (46), followed by *GATA1* (13) and *GFI1B* (10), with a limited number of genes with more than one TF motif predicted (Figure 7A). A network of the NSG predicted to be regulated by these NSTF was then constructed (Figure 7B). Interesting, 17 NSGs were predicted to be regulated by more than one NSTF, including *CD101*, *CERS4* and *SPHK1*, *CAMK1*, *IFIT1*, *MARCKS*, *TMEM92* and several unannotated genes. The NSTF gene *MXD1* was predicted to be regulated by both *GATA1* and *GFI1B*. Interestingly, several genes in this doubly-regulated group have been annotated to interact with other members of this group, including *ACSL1* (interaction partner: *ACSL4*), *CERS4* (interaction partner *SPHK1*), and *BST1* (two interaction partners: *ADAM18* and *C4BPA*) (Figure 7B). The latter interaction network is interesting as three other predicted NSTF target genes interact with BST1, indicating that BST1 may be an important protein in neutrophil function. An additional interaction network was seen for *IFIT1* which interacts with predicted targets *ISG15* and *HERC5*, as well as non-enriched gene *IFIT2*. Finally, several other genes are in the diagram that were not shown to have enrichment of nearby NSTF motifs (such as *MMP9*, *PDLIM1*, *TIAM2* and *ARHGAP15*), but nevertheless are predicted to interact with other genes predicted to be transcriptionally regulated by NSTF (Figure 7B).

## Discussion

In this study, we report the first comprehensive integrative analysis of chromatin accessibility and transcriptome of neutrophils isolated from non-stimulated pigs to expand the epigenetic and transcriptomic annotations of this important porcine innate immune cell. We then combined RNA-seq and ATAC-seq data to link neutrophil transcriptomic with genome-wide accessible chromatin data to identify potential regulatory relationships controlling neutrophil-specific gene expression. In consequence, we identified porcine neutrophil networks based on chromatin accessibility combined with TF and potential target genes enriched in neutrophil expression that may regulate this specific porcine neutrophil transcriptional program.

The RNA analysis of porcine neutrophils demonstrated these cells have a very different transcriptional pattern compared to previously reported porcine mononuclear cell transcriptomes (Herrera-Uribe et al., 2021) including three types of T cells (CD4^−^CD8^+^, CD4^+^CD8a^−^, CD8^+^CD4^+^, γδT-cells), NK and two types of B cells. However, the RNA expression patterns were more similar to the monocytes/DC population reported in that study. Further, the GO terms associated with genes whose expression was highly enriched in neutrophils were characteristic of known neutrophil function and pathways.

Based on the hypothesis that correlated expressed genes are more likely to have a similar biological role than genes with diverse expression pattern (Mola et al., 2020), we identified co-expressed gene clusters across the nine types of porcine immune cells mentioned above. Initially, we identified a myeloid cluster (for neutrophils and monocytes/DC), and by increasing the stringency, we identified a list of NSGs co-expressed specifically in neutrophils (SEG and co-expressed genes). The NSGs and myeloid dataset lists published here might be utilized in comparative analyses of neutrophils across species, to predict neutrophil content in whole blood via machine learning methods (Maslova et al., 2020).

Within the co-expressed neutrophil specific genes, it was interesting to identify a number of genes encoding TF. In fact, multiple TF with known roles within the innate immune system were found, especially TFs with myeloid and neutrophil functions given that both cell types are differentiated from the same bone marrow-residing precursor cell (Mehta et al., 2014). The well-known examples are essential TFs for myeloid and neutrophil differentiation such as *SPI1* (which encodes PU.1), *CEPA/B*, *GATA1*, *KLF5* and *GFI1B* (Bjerregaard et al., 2003; Duan and Horwitz, 2003; Shahrin et al., 2016; Denholtz et al., 2020; Khoyratty et al., 2021). Red and white blood cell differentiation is driven by GATA1 and PU.1, which act by mutual repression in the myeloid lineage (Nerlov et al., 2000). Recently, Fischer and others demonstrated that PU.1 drives an inhibitor epigenetic program in neutrophils which prevent the induction of an excessive innate immune response (Fischer et al., 2019).

*GFI1B*, a porcine NSTF found in our study has been shown to be an essential TF for neutrophil differentiation in mice; it is also necessary for the formation of both erythroid and megakaryocytic lineages in knockout mice (Saleque et al., 2002; Vassen et al., 2007). In human and mice, *GFI1B* mutations are linked to severe neutropenia and platelet shape, number, and function (Stevenson et al., 2013; Anguita et al., 2017; Geissler et al., 2018). The molecular function of *GFI1B* is to repress the expression of target genes (Vassen et al., 2005). *GFI1B* binds to the DNA, recruiting histone deacetylases and demethylases, which decrease gene expression (van der Meer et al., 2010). Another fundamental TF for neutrophil differentiation and commitment is *KLF5*, which was also found as a NSTF in our study. *In vivo* studies have revealed that loss of *KLF5* expression was associated with attenuated neutrophil differentiation (Shahrin et al., 2016). Additionally, reduction of *KLF5* expression was also reported in acute myeloid leukemia cancer (Humbert et al., 2011), where the neutrophils counts are also reduced (Zhang et al., 2021). Our approach further identified TFs highly expressed in porcine neutrophils and in other immune cell types. For example, *HHEX* and *FOSL2*, which in concordance with previous studies are highly expressed in neutrophils, B cells (Laidlaw et al., 2020) and NK cells (Grassi et al., 2018; Li et al., 2020), respectively. Additionally, we were able to find MSTFs such as *SPI1*, *CEBPB* and *CEBPA*, which also have been reported as highly expressed in human and mice myeloid lineages (Rosenbauer and Tenen, 2007; Mildner et al., 2017; Braun et al., 2019). Overall, these study results suggest a group of NSTFs in pig that have functional roles in the granulocyte lineage in other species such as mouse and human and therefore confirm the value of the porcine NSTFs list identified in this study.

We observed global chromatin accessibility regions that are comparable to those reported in mice (Fischer et al., 2019). Although ATAC-seq studies have been performed on human neutrophils, the depth of this analysis compared to our study was unclear as the number of total peaks in non-stimulated human neutrophils were not reported (Mistry et al., 2019; Ram-Mohan et al., 2021). In this study, we have detected slightly more accessible regions compared to those detected in mouse neutrophils. Khoyratty, et al., 2021 reported 47,164 ATAC-seq peaks across different murine neutrophil developmental stages (Khoyratty et al., 2021; Fischer et al., 2019) found around 20,000 ATAC-seq peaks (Fischer et al., 2019), in non-stimulated mouse neutrophils. Chromatin spatial and peak genomic distribution was similar to those reported in human neutrophils under non-stimulated conditions (Chen et al., 2016; Ram-Mohan et al., 2021). Interestingly, neutrophils plasticity has been attributed to changes in the chromatin structure, and the openness and closeness of the TFBM are responsible for the specific neutrophil response against a specific stimulus (Chen et al., 2016; Ram-Mohan et al., 2021). Ram-Mohan et al., 2021 identified specific chromatin changes in human neutrophils against different toll-like receptor (TLR) activators and *E. coli*, suggesting that highly differentiated neutrophils could modify their chromatin to affect transcriptional changes (reviewed in Rosales, 2018). Taken together, we provided for the first time the chromatin accessibility patterns in porcine neutrophils under non-stimulated conditions. Identifying the accessible chromatin regions and chromatin states with different pathogens or different TLR mimics in these cells would further evaluate neutrophil plasticity at the epigenomic and transcriptomic level in the pig.

By the integration of transcriptomic and chromatin accessibility data from biological replicate samples, we developed more detailed information on the transcriptional mechanics controlling gene expression in porcine neutrophils. First, we investigated the correlation between the promoter accessibility of a gene and its respective expression. We identified a significant positive correlation, which showed consistent correlation for all expressed genes and SEG. However, there were genes that showed an opposite pattern of promoter accessibility (no peak detected around promoter region) and expression (expressed in porcine neutrophils). Different studies have shown that low (or high) gene expression is not always associated with a lack of chromatin accessibility (Hughes et al., 2017; Su et al., 2017; Rajbhandari et al., 2018). As well, accessible chromatin regions around gene promoters are not always associated with expressed genes or regions marked with active histone modifications (Ackermann et al., 2016; Daugherty et al., 2017). Additionally, incomplete promoter annotation, especially in farm animals (Kern et al., 2021), could affect the correlation between accessible chromatin regions and gene expression.

We used recently published DNA methylation data from the same porcine neutrophil sample (and other eight porcine immune cells, see methods) (Corbett et al., 2022) to interrogate the relationship between DNA methylation levels, gene expression and chromatin accessibility. The integration of these data revealed that promoter regions with accessible chromatin exhibited low methylation, and highly neutrophil-enriched genes had a strong correlation with low methylated DNA in different genomic features, particularly with promoter regions. This negative association between DNA methylation, gene expression and chromatin accessibility have been observed in other immune cells and tissues (Pan et al., 2021; Roy et al., 2021; Corbett et al., 2022; Liu et al., 2022). Interestingly, only 119 of 832 SEG in neutrophils had LMRs around promoters in comparison with the eight porcine immune cells mentioned above. This suggest that highly expressed genes in neutrophils are likely controlled by a mechanism at least partially independent of DNA methylation, as it has been shown in other studies that DNA methylation and chromatin accessibility do not always follow an inverse correlation with gene expression (Lim et al., 2017; Spainhour et al., 2019). This result suggests that methylation and chromatin accessibility are two of multiple levels of transcriptional regulation (Thurman et al., 2012) for the porcine neutrophil transcriptome. Overall, while these data suggest that gene expression can be explained by chromatin accessibility around promoter regions, a further integrated analysis including more epigenetic data such as histone modifications and chromatin interaction data could complement current understanding regarding gene regulation in the pig. Such future work could also pay special attention to non-coding regions, which also shape gene expression patterns through enhancers and non-coding RNA activity (Chen et al., 2017; Panigrahi and O'Malley, 2021) as demonstrated in human neutrophils (Ecker et al., 2017).

As a main objective of this study was to identify the specific transcriptional network in porcine neutrophils according to gene expression and chromatin accessibility patterns, we also performed a TFBM enrichment analysis (using NSTF) on chromatin accessible regions around promoters of NSGs to predict networks of TF and their putative neutrophil specific target genes. Indeed, this approach identified several known lineage-specific TFs such as *GATA1*, *KLF5*, *MXD1* and *GFI1B* that are likely involved in maintaining neutrophils commitment and essential functions associated with those commitments. All these NSTF have been found expressed in other human mature neutrophil datasets (Monaco et al., 2019) and in early committed mice neutrophil progenitors (Kwok et al., 2020).

The combined analysis revealed that *GATA1* was predicted to bind the promoter region of the principal neutrophil-attracting chemokine and major mediator of the inflammation, *CXCL8* (Metzemaekers et al., 2020b). As well as *KLF5* was predicted to interact with promoters of *ISG15* (IFN-stimulated gene 15) and *IFIT1* (IFN-induce protein with tetratricopeptide repeats 1) (also predicted target of *GATA1*) and *CCR3* that are involved in the antiviral responses of neutrophils as a frontline of the innate immune system (Tamassia et al., 2008; Rudd et al., 2019). The putative target genes *IFIT1* and *ISG15* are also induced in response to bacteria in neutrophils (Xie et al., 2020). In general, our integrative approach identified TFs and promoter accessible regions of target genes within NSTF. However, the predictions that these TFs regulate the genes with motif-enriched promoters enriched would need to be validated with genetic/biochemical experiments to provide more insight into the regulatory mechanisms of gene regulation in porcine neutrophils.

Interestingly, we identified a TF that has not been widely studied in neutrophils, MAX dimerization protein 1 gene (*MXD1*), as a NSTF potentially involved in neutrophil regulatory networks. Interestingly, *MXD1* was predicted to be controlled by other two NSTFs, *KLF5* and *GFI1B*. Lummertz da Rocha, E et al., 2018 identified increase of the *MXD1* gene expression in the later stages of mature neutrophil differentiation from mouse bone marrow scRNA-seq data (Lummertz da Rocha et al., 2018). Further, *MXD1* expression was significantly associated with the low proportion of neutrophils in tumor immune microenvironment data which included information from 22 cell types (Du et al., 2021). In the Lummertz da Rocha et al., 2018 study, a gene regulatory network analysis linked *MMP8*, *MMP9*, *RETNLG* and *CD52* genes as predicted targets of *MXD1*. In our experimental approach using ATAC-seq data, we were able to identify *MMP9* (which mediates neutrophil migration in infection (Bradley et al., 2012)) as a SEG that interact with other NSGs (*CXCL8*, *NCF1* and *TIMP3*) in porcine neutrophils, but we did not detect a predicted binding motif for NSTFs in the *MMP9* TSS-OCR.

Some of the genes displayed in the specific transcriptional network in porcine neutrophils interacted with other NSGs and interact with other predicted NSTF target genes. All these multiple interactions in the network suggest a collaborative and important function of these genes in neutrophils. For example, BST1 (CD157) protein is highly expressed on the surface of most human circulating neutrophils and plays a fundamental role in neutrophil adhesion and migration (Funaro et al., 2004; Ortolan et al., 2006). Additionally, we identified a group of genes that interact with other predicted transcriptional regulated genes, although they were not predicted to be transcriptionally regulated by any of NSTF. Two of these genes could illustrate the non-activated state of the neutrophils we used in our study, for example, *PDLM1* and *ARHGAP15*. The PDLIM1 protein, a member of the LIM domain-binding protein family, negatively regulates NF-κB-activation in the cytoplasm by sequestration of p65 (subunit of NF-κB), suppressing NF-κB translocation to the nuclei (Ono et al., 2015). ARHGAP15, a GTPase of the Rac family is also a negative regulator of neutrophil function (Costa et al., 2011). Costa et al. (2011) observed in mice lacking *ARHGAP15* an increase of neutrophil recruitment to the site of infection, reducing systemic inflammation and improving mice survival. They also found enhancement of chemotactic activity, straighter directional migration, amplified reactive oxygen species production, increase of phagocytosis and bacterial killing *in vitro* in cells lacking *ARHGAP15*. Together, our study identified a gene network in porcine neutrophils that contains several well-known essential genes for neutrophil functions as well as potential novel gene candidates that could expand the understanding of the neutrophil, especially in pig.

The epigenetic (histone ChIP, ATAC-seq and DNA methylation analyses) and transcriptional data (RNA-seq) provided by the FAANG project is contributing to the identification of regulatory regions in the porcine genome (Giuffra et al., 2019; Herrera-Uribe et al., 2021; Kern et al., 2021; Pan et al., 2021; Corbett et al., 2022). Such data is very powerful to detect regulatory regions, but it is time-intensive and comes with significant cost. Our strategy using only RNA-seq and ATAC-seq may be more generally applied to identify many of these elements and so improve the pig genome annotation of other porcine circulating immune cells requiring many biological states. These states would include resting and different immune state conditions such as response to bacterial and viral infections or immune-related diseases. We also demonstrated how these data can be utilized to inform researchers to link TFs, open chromatin, and gene expression to predict the interactions of these regulatory elements to form transcriptional networks. Thus, the integrative analysis presented in this manuscript will be a useful tool to extend the epigenetic and transcriptomic annotations to the other porcine immune cell types.

## Data Availability

The datasets presented in this study can be found in online repositories. The names of the repository/repositories and accession number(s) can be found below: https://www.ebi.ac.uk/ena, PRJEB57595; https://www.ebi.ac.uk/ena, PRJEB51699.

## Footnotes

1
https://broadinstitute.github.io/picard/

2
https://hbctraining.github.io/Intro-to-ChIPseq/lessons/05_peak_calling_macs.html

3
http://homer.ucsd.edu/homer/ngs/annotation.html

4
http://homer.ucsd.edu/homer/

5
https://www.encodeproject.org/atac-seq/

## Abbreviations

ACD, Acid Citrate Dextrose; ACK, Ammonium-Chloride-Potassium; ATAC-seq, Assay for Transposase Accessible Chromatin; DC, Dendritic Cells; DGE, Differential Gene Expression; DMSO, Dimethyl sulfoxide; FAANG, Functional Annotation of Animal Genomes; FRiP, Fraction of reads in called peak regions; GO, Gene oOntology; HBSS, Hanks’ Balance Salt Solution; HEGs, Highly Enriched Genes; IGV, Integrative Genomics Viewer; LMR, Low Methylated Region; MCODE, Molecular Complex Detection; MSTF, Myeloid Specific Transcription Factor; NSGs, Neutrophil Specific Genes; NSTF, Neutrophil Specific Transcription Factor; PBMCs, Peripheral Blood Mononuclear Cells; PCA, Principal Component Analysis; PPI, Protein-Protein Interaction; OCRs, Open Chromatin Regions; RT-qPCR, Reverse Transcriptase Polymerase Chain Reaction; TLR, Toll-Like Receptor; TF, Transcription Factor; TFBM, Transcription Factor Binding Motifs; TMM, Trimmed Mean of M Values; TPM, Transcript Per Million; TSS, Transcription Start Site; SEGs, Significantly Enriched Genes.
