# Supplementary material for: Integrative profiling of gene expression and chromatin accessibility elucidates specific transcriptional networks in porcine neutrophils
Source: Front Genet. 2023 May 23;14:1107462. doi: 10.3389/fgene.2023.1107462 (PMC10242145; doi:10.3389/fgene.2023.1107462)
Supplement: Supplementary file 5 [file Image1.pdf]

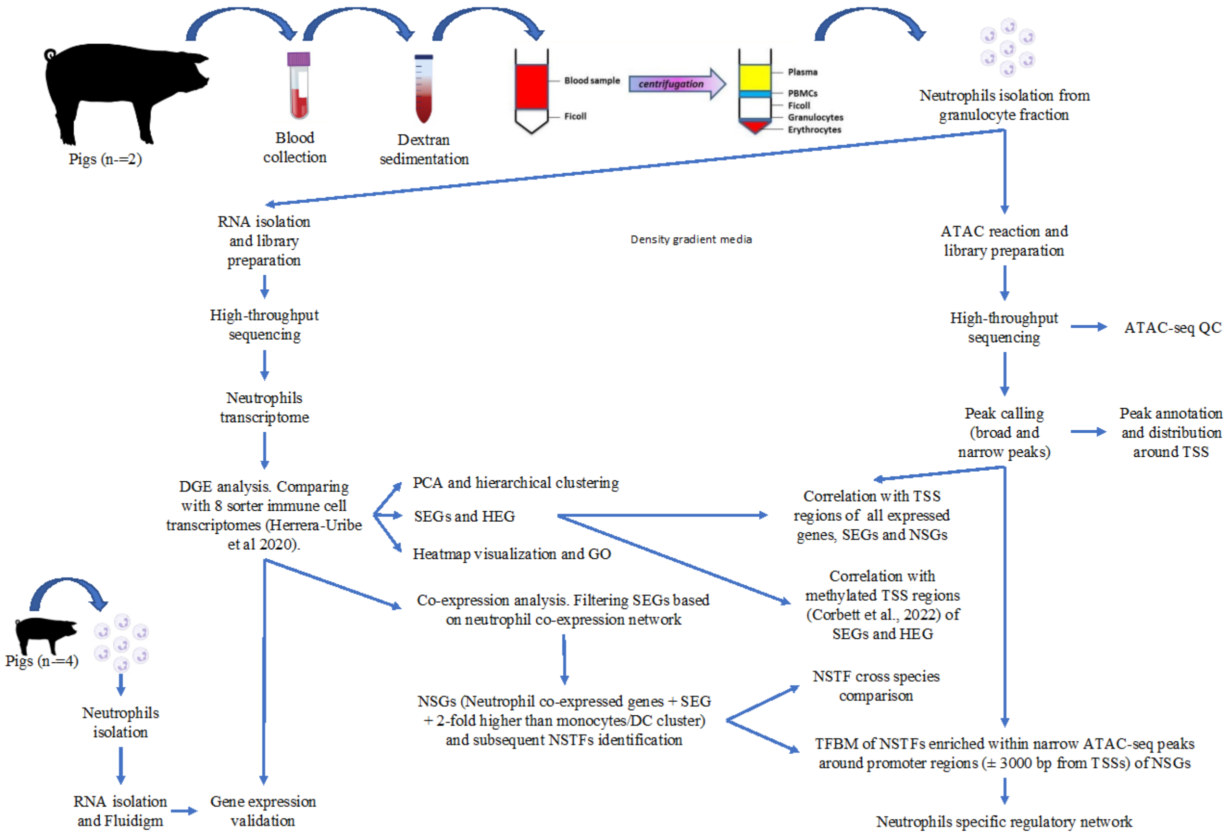

Supplementary Figure 1. Experimental flowchart. ATAC: Assay for Transposase Accessible Chromatin, DC: , DGE: Differential gene expression, GO: Gene ontology, HEG: Highly enriched genes, NSGs: Neutrophil specific genes, NSTF: Neutrophil specific transcription factor, PCA: Principal component analysis, QC: Quality Control, SEG: Significantly Enriched Genes, TFBM: Transcription factor binding motifs, TSS: Transcription start site.

A)

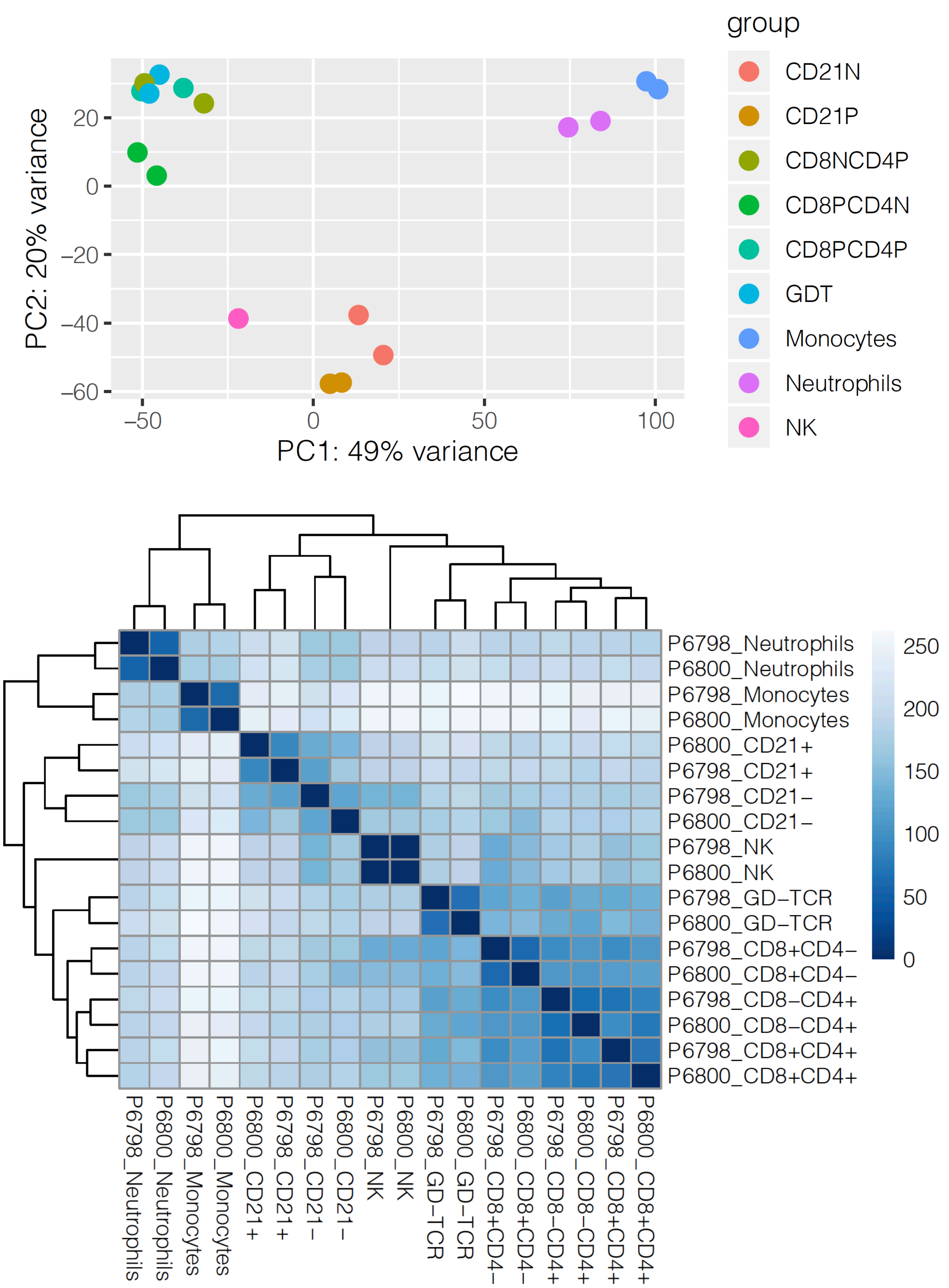

Supplementary Figure 2. Transcriptional expression patterns of immune cells are distinct and cluster more by progenitors. (**A**) Principal component analysis of transformed RNA-seq reads counts for whole transcriptomes. Axis indicate component scores. (**B**) Heat map depicting hierarchical clustering of sample-to-sample distance. Gene expression for whole transcriptomes were used to calculate sample to sample Euclidean distance (color scale) for hierarchical clustering.

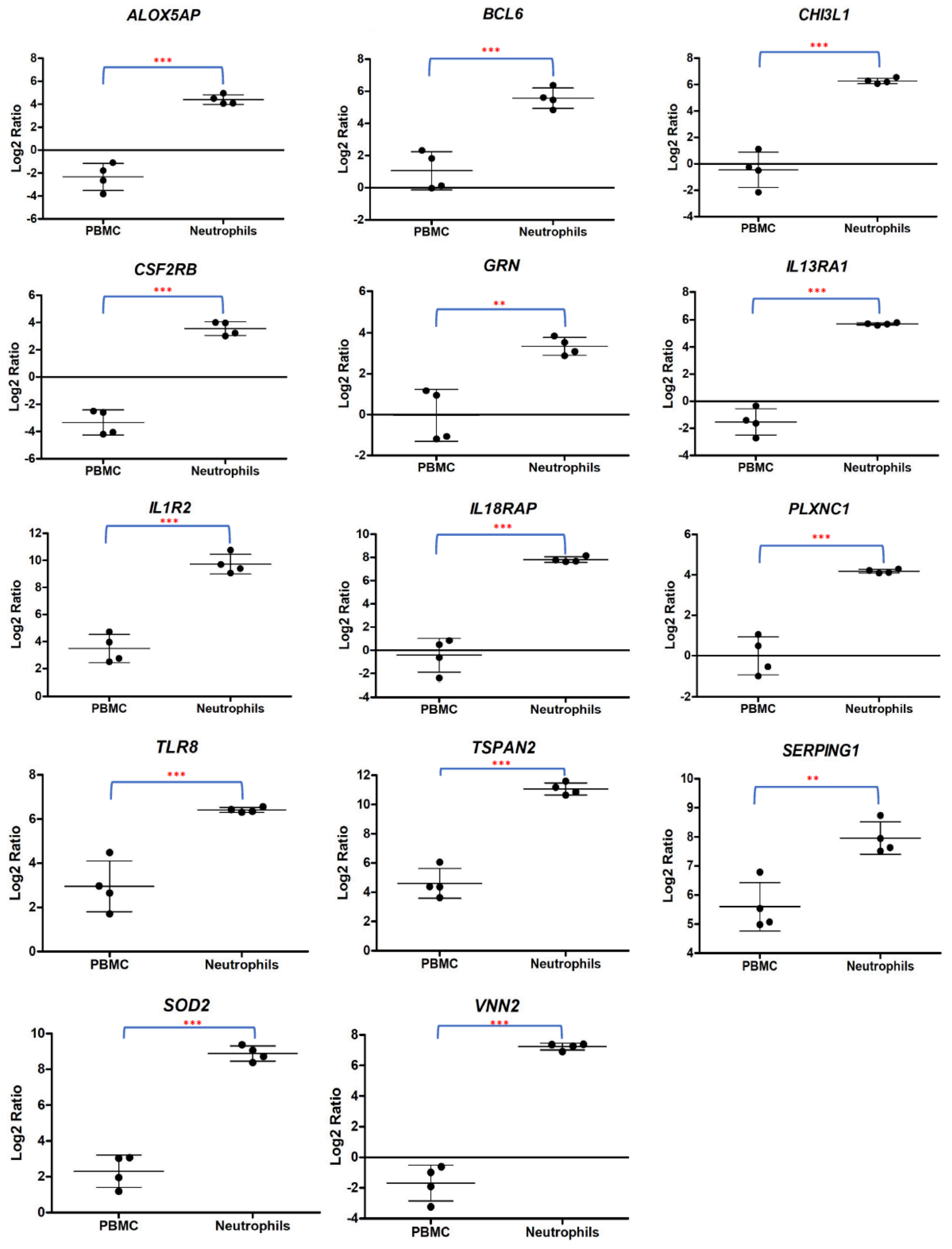

Supplementary Figure 3. Gene expression of neutrophil enriched genes determine by Fluidigm HD Biomark system. Graphs showing Log2ratios of selected genes in PBMCs and isolated neutrophils.

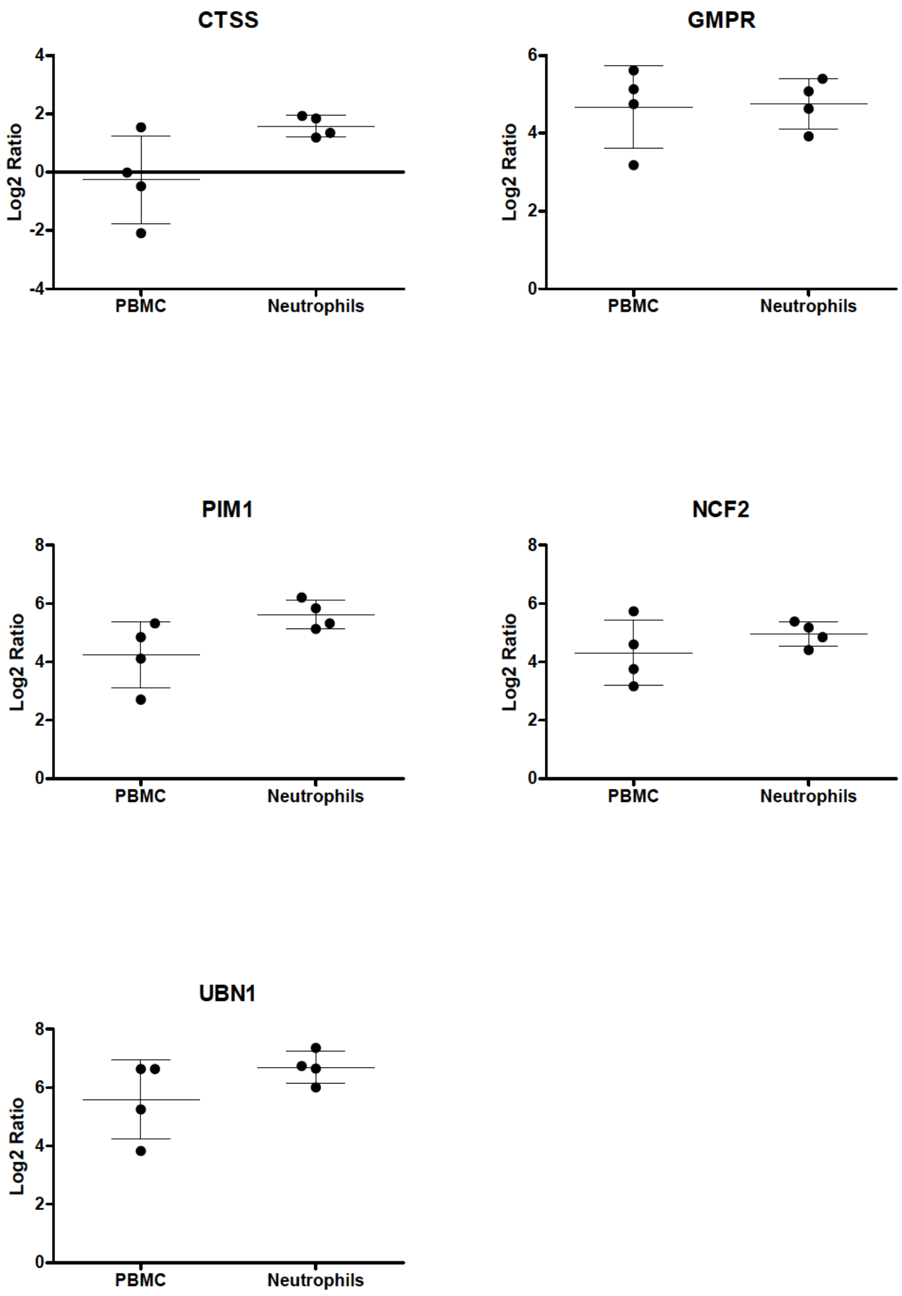

Supplementary Figure 4. Gene expression of non-neutrophil enriched genes determine by Fluidigm HD Biomark system. Graphs showing Log2ratios of selected genes in PBMCs and isolated neutrophils.

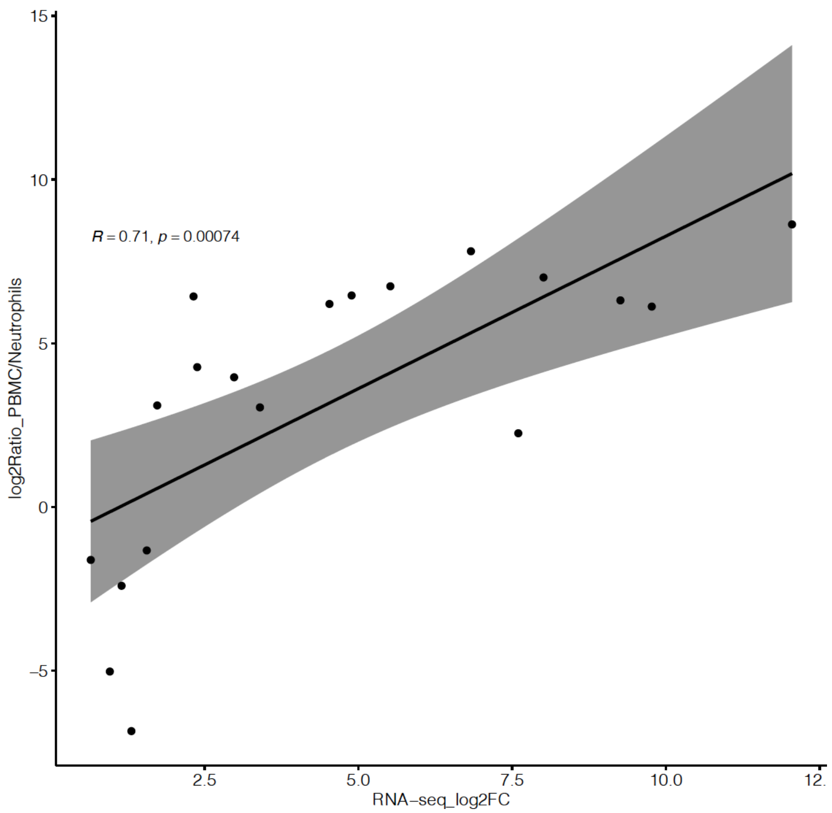

Supplementary Figure 5. Bulk RNA Fluidigm HD Biomark system correlation. Pearson correlation of log2ratios of selected genes between RNA-seq and Fluidigm HD Biomark system.

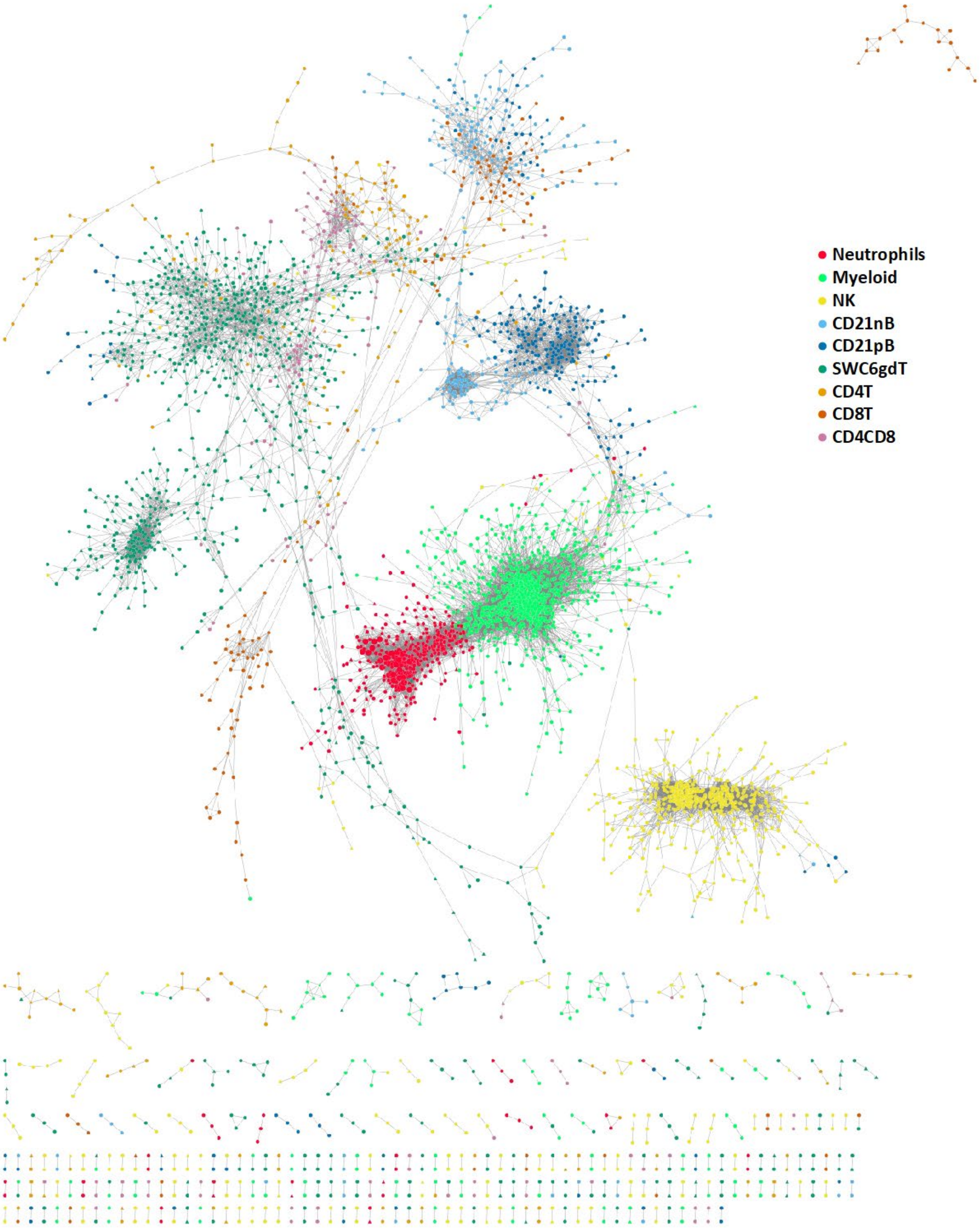

Supplementary Figure 6. Co-expression network (r>0.95) across 9 porcine cell types. The node (gene) colors indicate the cell type that showed the highest expression among cell types. The big nodes indicate the genes that showed significantly higher expression in neutrophils than all other cell types. The triangle nodes indicate the genes that encode transcription factors in pigs.

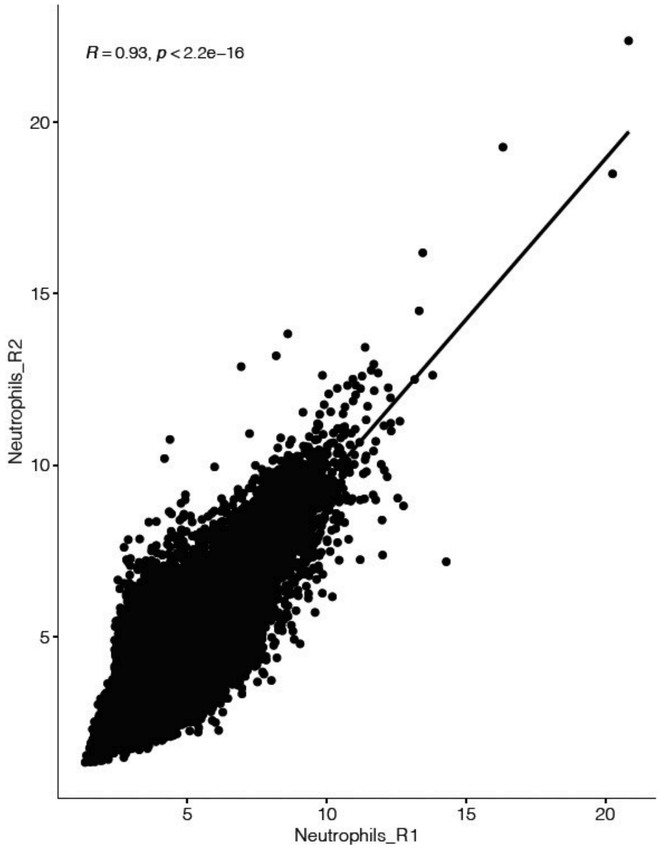

Supplementary Figure 7. Pearson correlation of ATAC-seq signal intensity between two neutrophil ATAC-seq replicates.

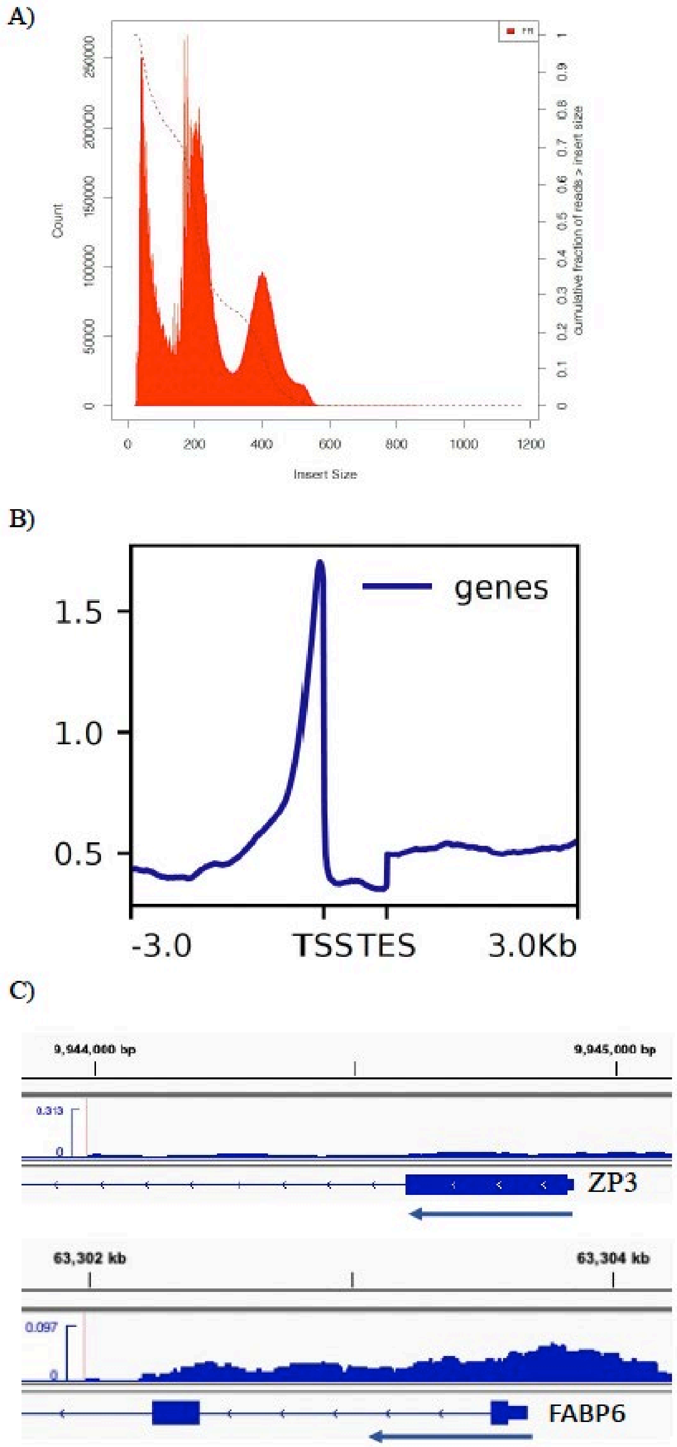

Supplementary Figure 8. ATAC-seq analysis of porcine neutrophils. **A**) Distribution of ATAC-seq data fragment length from one neutrophil replicate. Fragment size distribution plot shows enrichment around 100-200, indicating nucleosome-free region and mono-nucleosome-bound fragments. **B**) TSS enrichment plot from one neutrophil replicate shows that nucleosome-free fragments are enriched at TSS. **C**) Peak visualization using the Integrative Genomics Viewer (IGV) of low or non-expressed genes in porcine neutrophils showed low fold enrichment of accessible chromatin compared to SEGs (ZP3 TPM: 1 and FAB6: no detected).

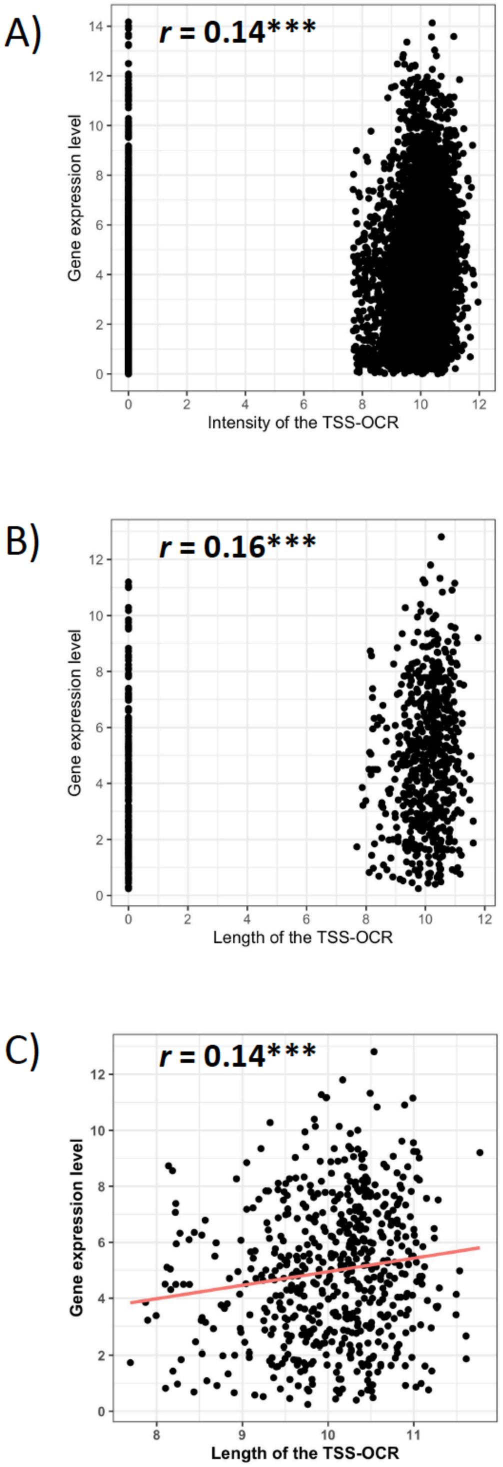

Supplementary Figure 9. Relationship of gene expressions with the length of the TSS-OCRs for all genes (A), neutrophils enriched genes (C), and relationship of gene expressions with the length (C) of the TSS-OCRs for genes that had at least 1 TSS-OCR among genes whose expressions were enriched for neutrophils. TSS-OCR, open chromatin regions in ±3 kb from the transcription start site (TSS).

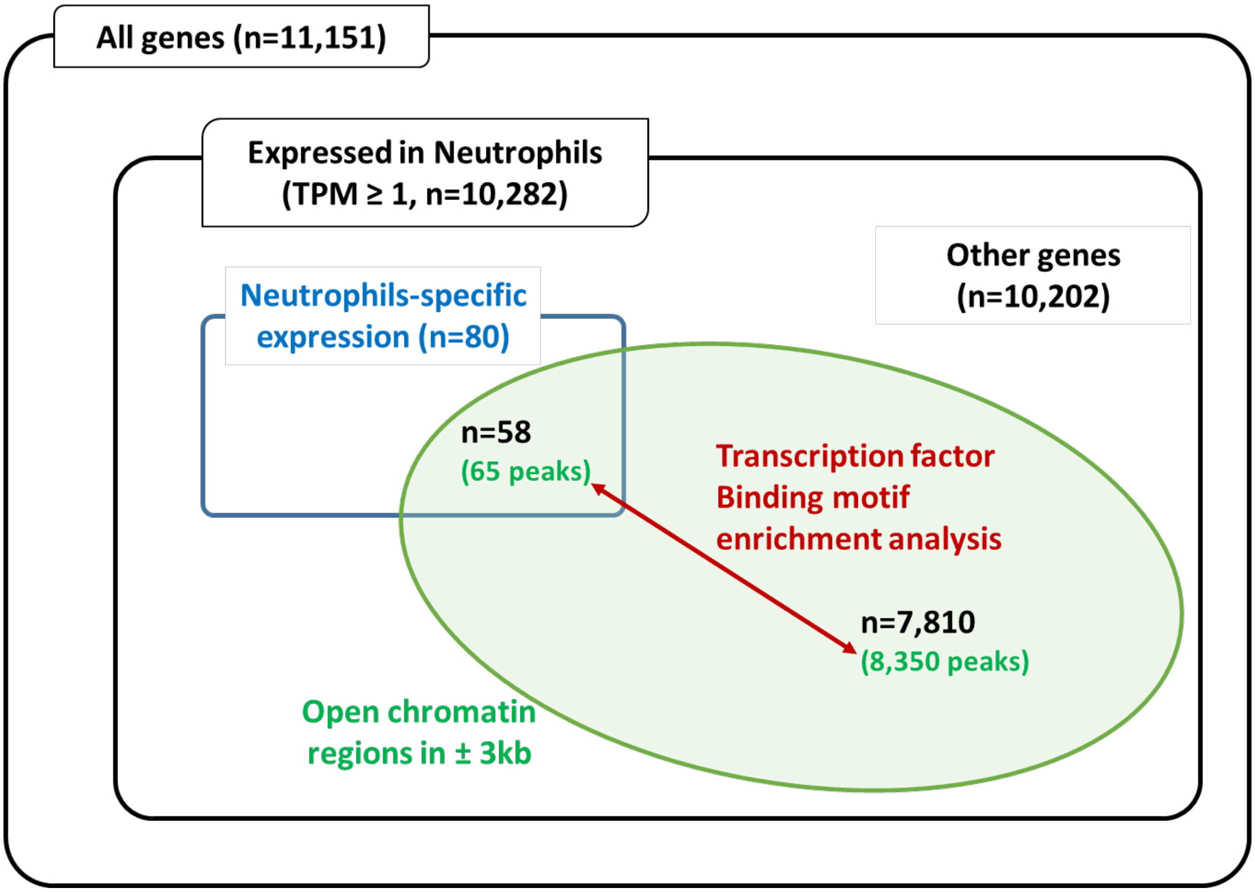

Supplementary Figure 10. **N**umber of ATAC-seq peaks found in neutrophils specific genes and number of ATAT-seq used for transcription factor binding motif analysis (only ATAC-seq peaks around ±3 kb from the transcription start site were counted).
